# Highly Reduced Ruthenium Carbide Carbonyl Clusters: Synthesis, Molecular Structure, Reactivity, Electrochemistry, and Computational Investigation of [Ru_6_C(CO)_15_]^4–^

**DOI:** 10.1021/acs.inorgchem.3c01711

**Published:** 2023-08-30

**Authors:** Cristiana Cesari, Marco Bortoluzzi, Tiziana Funaioli, Cristina Femoni, Maria Carmela Iapalucci, Stefano Zacchini

**Affiliations:** † Dipartimento di Chimica Industriale “Toso Montanari”, Università di Bologna, Viale Risorgimento 4, 40136 Bologna. Italy; ‡ Dipartimento di Scienze Molecolari e Nanosistemi, Ca’ Foscari University of Venice, Via Torino 155, 30175 Mestre (Ve), Italy; § Dipartimento di Chimica e Chimica Industriale, Università di Pisa, Via G. Moruzzi 13, 56124 Pisa, Italy

## Abstract

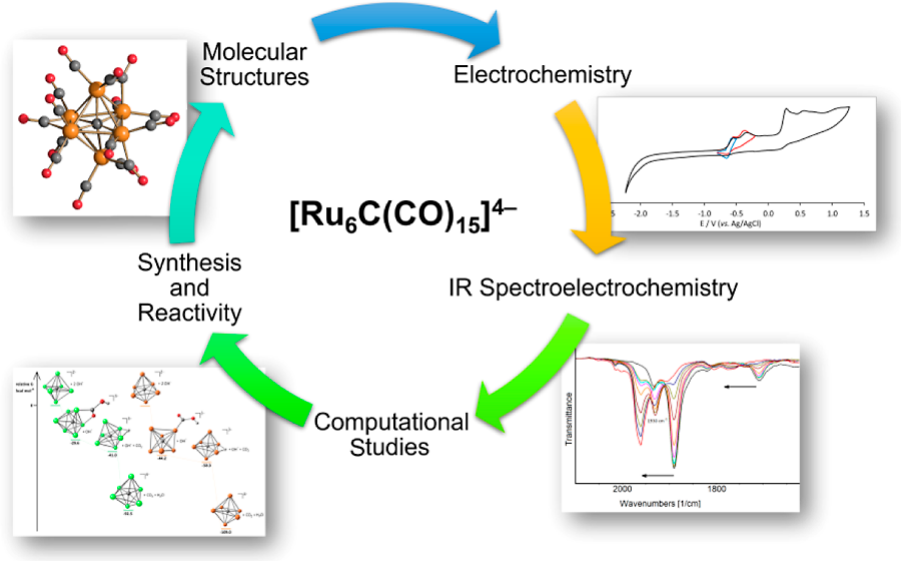

The reaction of [Ru_6_C(CO)_16_]^2–^ (**1**) with NaOH in DMSO resulted in the formation of a highly reduced [Ru_6_C(CO)_15_]^4–^ (**2**), which was readily protonated by acids, such as HBF_4_·Et_2_O, to [HRu_6_C(CO)_15_]^3–^ (**3**). Oxidation of **2** with [Cp_2_Fe][PF_6_] or [C_7_H_7_][BF_4_] in CH_3_CN resulted in [Ru_6_C(CO)_15_(CH_3_CN)]^2–^ (**5**), which was quantitatively converted into **1** after exposure to CO atmosphere. The reaction of **2** with a mild methylating agent such as CH_3,_I afforded the purported [Ru_6_C(CO)_14_(COCH_3_)]^3–^ (**6**). By employing a stronger reagent, that is, CF_3_SO_3_CH_3_, a mixture of [HRu_6_C(CO)_16_]^−^ (**4**), [H_3_Ru_6_C(CO)_15_]^−^ (**7**), and [Ru_6_C(CO)_15_(CH_3_CNCH_3_)]^−^ (**8**) was obtained. The molecular structures of **2–5**, **7**, and **8** were determined by single-crystal X-ray diffraction as their [NEt_4_]_4_[**2**]·CH_3_CN, [NEt_4_]_3_[**3**], [NEt_4_][**4**], [NEt_4_]_2_[**5**], [NEt_4_][**7**], and [NEt_4_][**8**]·solv salts. The carbyne–carbide cluster **6** was partially characterized by IR spectroscopy and ESI-MS, and its structure was computationally predicted using DFT methods. The redox behavior of **2** and **3** was investigated by electrochemical and IR spectroelectrochemical methods. Computational studies were performed in order to unravel structural and thermodynamic aspects of these octahedral Ru–carbide carbonyl clusters displaying miscellaneous ligands and charges in comparison with related iron derivatives.

## Abstract 2

A highly reduced ruthenium-carbide carbonyl cluster was prepared and employed as a platform for the synthesis of further Ru_6_C clusters, which were investigated by a combination of chemical, spectroscopic, structural, electrochemical, spectroelectrochemical, and computational methods.

## 1 Introduction

Octahedral Ru_6_C(CO)_17_ represented the first structurally characterized metal carbonyl cluster containing a fully interstitial carbide atom.^1−3^ Since then, the number of such compounds has considerably grown, including other transition metals, such as Fe, Os, Co, Rh, Ni, as well as heterometallic and heteroleptic clusters.^4−11^ The stabilizing effect of interstitial main group atoms, including carbides, is nowadays well established.^12−16^ Moreover, metal carbide carbonyl clusters have served as models of the reactivity of carbon atoms on metal surfaces and metal nanoparticles, greatly contributing to the development of the cluster–surface analogy and the study of the Fischer–Tropsch reaction.^17−24^ More recently, metal carbide carbonyl clusters have attracted renewed interest in view of their application in electrocatalytic processes, such as hydrogen evolution reaction (HER) and CO_2_ reduction.^25−29^ In addition, the discovery of the presence of a carbide atom within the Fe–S cluster of nitrogenase enzymes^30−33^ also prompted some studies on iron carbide carbonyl clusters.^34−38^

In a recent study, Chaudret et al. employed Ru–carbide carbonyl clusters as models for the formation of carbides on Ru nanoparticles during CO hydrogenation.^39^ Heterometallic Ru-based carbide carbonyl clusters have been used as precursors for the preparation of heterogeneous catalysts supported on mesoporous MCM41 and other solid supports.^40−45^ Anionic Ru carbide carbonyl clusters are valuable precursors for the synthesis of heterometallic clusters and self-assembly of molecular materials.^46−49^

Three octahedral homoleptic Ru carbide carbonyl anions are known: [Ru_6_C(CO)_16_]^2–^, [HRu_6_C(CO)_16_]^−^, and [HRu_6_C(CO)_15_]^−^.^50−53^ The latter possesses 84 cluster valence electrons (CVE) rather than 86 CVE, as normally found in octahedral carbonyl clusters, including [Ru_6_C(CO)_16_]^2–^ and [HRu_6_C(CO)_16_]^−^.^54,55^ As it is shown here, it is likely that [HRu_6_C(CO)_15_]^−^ might be better reformulated as an electron precise 86 CVE trihydride, that is, [H_3_Ru_6_C(CO)_15_]^−^.

It was recently reported that treatment of [Fe_6_C(CO)_16_]^2–^ with NaOH in DMSO or Na/naphthalene in THF afforded a highly reduced [Fe_6_C(CO)_15_]^4–^ anion.^56^ Herein, we report the synthesis and structural characterization of the Ru analogue [Ru_6_C(CO)_15_]^4–^, as well as the study of its reactivity that resulted in further new Ru_6_C carbide carbonyl clusters. All the new compounds have been spectroscopically characterized and their structures determined by single-crystal X-ray diffraction (SC-XRD). The redox chemistry of the highly reduced [Ru_6_C(CO)_15_]^4–^ cluster was investigated by electrochemical and IR spectroelectrochemical methods. All the new findings were further supported by computational studies employing DFT methods.

## 2 Results and Discussion

### 2.1 Synthesis and Molecular Structure of [Ru_6_C(CO)_15_]^4–^ and [HRu_6_C(CO)_15_]^3–^

The reaction of [Ru_6_C(CO)_16_]^2–^ (**1**) with NaOH in DMSO resulted in the formation of [Ru_6_C(CO)_15_]^4–^ (**2**), which was isolated as [NEt_4_]_4_[**2**]**·**CH_3_CN crystals suitable for SC-XRD after working up of the reaction mixture (see Section 4 for details). As previously reported for the synthesis of [Fe_6_C(CO)_15_]^4–^,^56^ formation of **2** is likely to proceed via nucleophilic attack of OH^–^ to a coordinated CO of **1**, followed by reduction of the cluster and elimination of CO_2_ and H_2_O according to eq 1. This is a well-documented reaction in the chemistry of metal carbonyl clusters.^7,8,16^ This mechanism has been further supported by DFT studies (see Section 2.4).
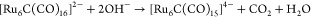
1

**Scheme 1:**
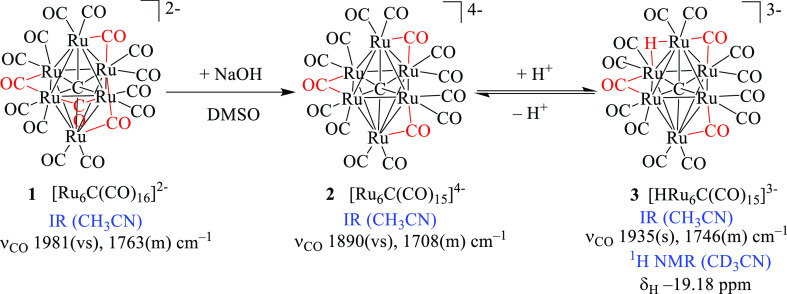
Synthesis of [Ru_6_C(CO)_15_]^4–^ (**2**) and [HRu_6_C(CO)_15_]^3–^ (**3**)

The reaction was easily monitored by IR spectroscopy (Scheme 1), since the ν_CO_ bands of **1** [ν_CO_ 1981(vs), 1763(m) cm^–1^, Figure S1 in the Supporting Information] were significantly red shifted after reduction to **2** [ν_CO_ 1890(vs), 1708(m) cm^–1^; Figure S2 in the Supporting Information]. Alternatively, **2** could be obtained by treating **1** with Na/naphthalene in THF. The tetra-anion **2** was readily protonated to [HRu_6_C(CO)_15_]^3–^ (**3**) (see below for its characterization and Figure S3 in the Supporting Information) and, often, the two compounds were obtained in mixture. They could be separated owing to their different solubility in organic solvents. Indeed, as [NEt_4_]^+^ salts, **3** was soluble in acetone and **2** in CH_3_CN. It must be noted that improved yields of **2** could be obtained by adding dropwise the crude DMSO reaction mixture to a solution of [NEt_4_]Br in H_2_O/^i^PrOH. In this way, [NEt_4_]_4_[**2**] precipitated immediately and the formation of **3** was limited. Conversely, addition of a saturated water solution of [NEt_4_]Br to the crude DMSO reaction mixture caused the precipitation of [NEt_4_]_4_[**2**] in a mixture with a significant amount of [NEt_4_]_3_[**3**].

**Figure 1:**
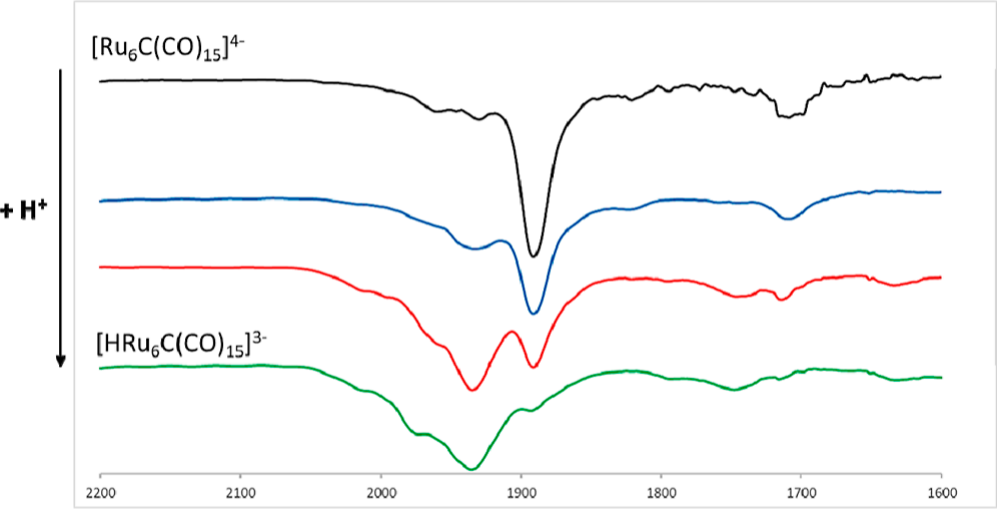
Reaction of **2** with HBF_4_·Et_2_O in CH_3_CN monitored by IR spectroscopy. IR spectra were recorded after the addition of 0.30 equiv each time.

Complete conversion of **2** into **3** could be achieved after treating the former with a stoichiometric amount of HBF_4_·Et_2_O as monitored by IR spectroscopy (Figure 1). The molecular structure of **3** was fully unraveled by SC-XRD as [NEt_4_]_3_[**3**] crystals. The hydride nature of **3** was confirmed by the presence of a singlet at δ_H_ −19.18 ppm in the ^1^H NMR spectrum in CD_3_CN (Figures S10 and S11 in the Supporting Information). Further addition of 1 mol equiv of HBF_4_**·**Et_2_O to **3** resulted in the formation of **1** as demonstrated by IR spectroscopy (Figure S4 in the Supporting Information). Similar results were obtained employing HCl**·**Et_2_O instead of HBF_4_**·**Et_2_O, except that, due to the stronger coordination capability of Cl^–^ compared to [BF_4_]^−^, some crystals of [NEt_4_][RuCl_3_(CO)_2_(CH_3_CN)_2_] were obtained as side products (Figure S21 in the Supporting Information).

**Figure 2:**
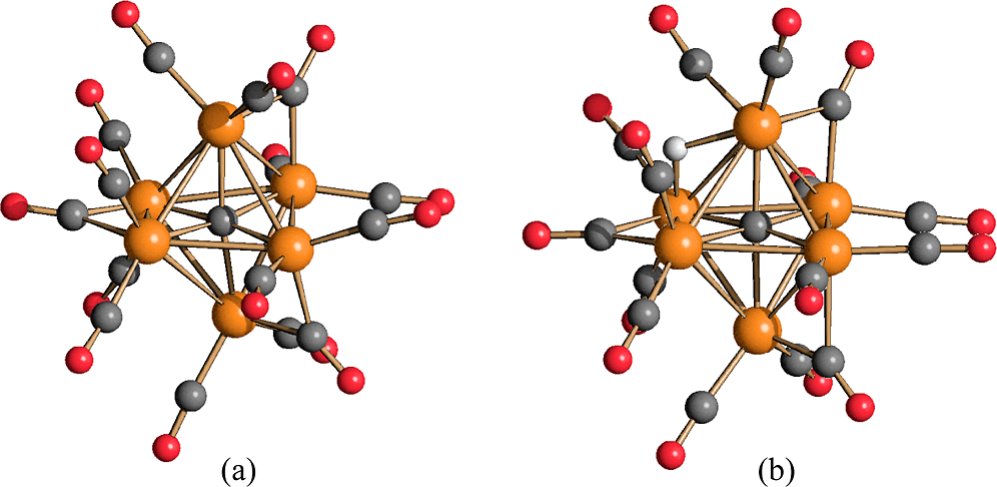
Molecular structures of (a) [Ru_6_C(CO)_15_]^4–^ (**2**) and (b) [HRu_6_C(CO)_15_]^3–^ (**3**) (orange Ru; red O; gray C; white H).

The molecular structures of **2** and **3** are both based on a carbide-centered Ru_6_C octahedron (Figure 2). There are 12 terminal carbonyls and, in addition, 3 μ-CO ligands are bridging 3 nonconsecutive Ru–Ru edges. Thus, each Ru atom is bonded to two terminal and one μ-CO ligand. The μ-H hydride ligand of **3** is located on a further Ru–Ru edge. The Ru–Ru and Ru–C_carbide_ distances of **2** and **3** are very similar (Table 1). They are also comparable to those reported for **1** and [HRu_6_C(CO)_16_]^−^ (**4**).^50−53^ This is probably the result of the compensation of two opposite effects: increasing the negative charge in the series **4**, **1**, **3**, **2** should lead to increased bonding distances, whereas decreasing the number of ligands in the same series should reduce steric hindrance and, thus, the bonding parameters.

**Table 1:** Bonding Contacts (Å) and Ligand Stereochemistry of [Ru_6_C(CO)_15_]^4–^ (**2**), [HRu_6_C(CO)_15_]^3–^ (**3**), and [H_3_Ru_6_C(CO)_15_]^−^ (**7**), Compared to [Ru_6_C(CO)_16_]^2–^ (**1**), [HRu_6_C(CO)_16_]^−^ (**4**), and Ru_6_C(CO)_17_

	Ru–Ru	Ru–C	t-CO	μ-CO	H
[Ru_6_C(CO)_15_]^4–^ (**2**)	2.703(4)–3.196(4) average 2.906(10)	2.029(2)–2.086(13) average 2.05(2)	12	3	
[HRu_6_C(CO)_15_]^3–^ (**3**)	2.774(3)–3.021(3) average 2.897(15)	2.00(3)–2.10(4) average 2.05(12)	12	3	μ
[H_3_Ru_6_C(CO)_15_]^−^ (**7**)	2.8082(12)–2.9857(12) average 2.888(4)	1.997(10)–2.072(10) average 2.04(2)	12	3	μ
[Ru_6_C(CO)_16_]^2–^ (**1**)^a^	2.8480(10)–3.0010(10) average 2.890(5)	2.038(2)–2.065(2) average 2.044(7)	12	4	
[HRu_6_C(CO)_16_]^−^ (**4**)^b^	2.8210(5)–2.9873(5) average 2.8897(17)	2.029(4)–2.051(4) average 2.044(7)	12	4	μ
Ru_6_C(CO)_17_^c^	2.834(3)–2.967(3) average 2.902(10)	2.015(5)–2.085(5) average 2.05(12)	16	1	

a From ref (50).

b From ref (51).

c From ref (2).

The new clusters **2** and **3**, as well as the previously reported **1** and **4**, possess 86 CVE, as expected for an octahedral cluster.^55^

### 2.2 Oxidation Reactions of [Ru_6_C(CO)_15_]^4–^

**Scheme 2:**
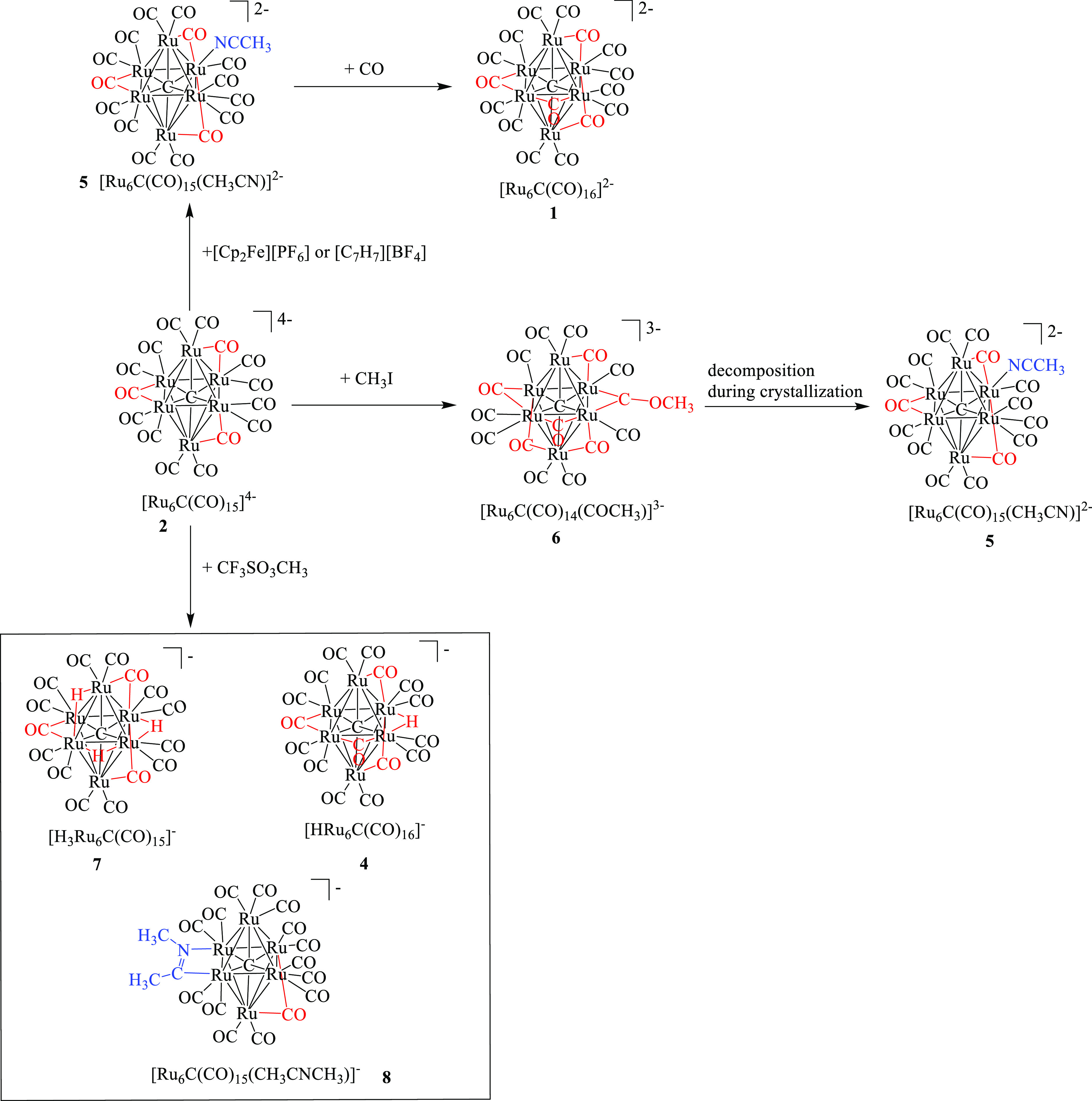
Oxidation Reactions of [Ru_6_C(CO)_15_]^4–^ (**2**) in CH_3_CN. All the species have been structurally characterized by SC-XRD except [Ru_6_C(CO)_14_(COCH_3_)]^3–^ (**6**), which was identified by spectroscopic methods (IR and ESI-MS) and its structure computationally determined.

The reactions of **2** in CH_3_CN with miscellaneous oxidizing and alkylating agents, that is, [Cp_2_Fe][PF_6_], [C_7_H_7_][BF_4_], CF_3_SO_3_CH_3_, and CH_3,_I have been studied (Scheme 2).

**Figure 3:**
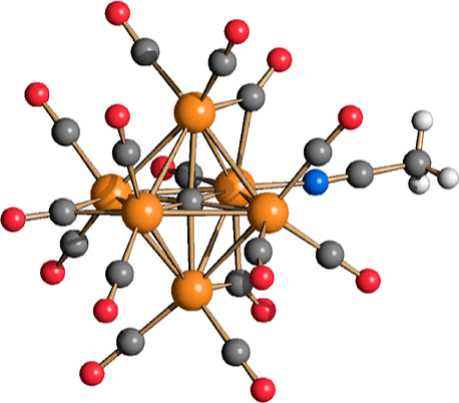
Molecular structure of [Ru_6_C(CO)_15_(CH_3_CN)]^2–^ (**5**) (orange Ru; red O; blue N; gray C; white H). Main bond distances (Å): Ru–Ru 2.7901(7)–2.9702(7), average 2.888(2); Ru–C_carbide_ 2.019(6)–2.056(6), average 2.042(15); Ru–N_CH3CN_ 2.075(6); C–N_CH3CN_ 1.126(9).

The chemical oxidation of **2** [ν_CO_ 1890(vs), 1708(m) cm^–1^] with [Cp_2_Fe][PF_6_] or [C_7_H_7_][BF_4_] in CH_3_CN resulted in a new species displaying ν_CO_ at 1963(s) and 1760(m) cm^–1^ (Figure S5 in the Supporting Information) as found during the electrochemical oxidation (see Section 2.3). This new species revealed to be [Ru_6_C(CO)_15_(CH_3_CN)]^2–^ (**5**), as demonstrated by SC-XRD analysis on its [NEt_4_]_2_[**5**] salt (Figure 3). [C_7_H_7_][BF_4_] revealed poor selectivity in the oxidation of **2** into **5** (yield 36%), due to the formation of side products such as **1**. Better yield (61%) was obtained using [Cp_2_Fe][PF_6_], for which the expected stoichiometric ratio, that is, 2 mol of [Cp_2_Fe][PF_6_] per mole of **2** was employed. Indeed, formation of **5** may be viewed as a two-electron oxidation of **2** with concomitant coordination of CH_3_CN to the cluster, as was electrochemically demonstrated.

Compound **5** was quantitatively converted into **1** [ν_CO_ 1981(vs), 1763(m) cm^–1^] after exposure to CO atmosphere, following replacement of the labile CH_3_CN ligand by the stronger CO ligand. The ν_CO_ bands of **5** absorb at slightly lower wavenumbers compared to **1**, in view of the stronger σ-donor ability of CH_3_CN compared to CO.

The molecular structure of **5** closely resembles that of **1**. The replacement of one terminal CO ligand with CH_3_CN causes a slight rearrangement of the CO ligands. Indeed, **5** displays three μ-CO ligands, and **1** contains 4 μ-CO ligands, whereas the remaining 12 carbonyls are in terminal positions in both the structures. The bonding parameters of the Ru_6_C cage are comparable to all the other clusters reported here.

The reaction of **2** with CH_3_I led to a purported methylated species [Ru_6_C(CO)_14_(COCH_3_)]^3–^ (**6**) as evidenced by IR, ^1^H NMR, and ESI-MS analyses (Figures S7, S8, S15, and S20 in the Supporting Information). Indeed, the IR spectrum of **6** [ν_CO_ 1939(vs), 1748 cm^–1^] is very similar to that of **3** [ν_CO_ 1935(s), 1746(m) cm^–1^], in view of the fact that both possess a 3– charge. Nonetheless, **6** does not show any hydride resonance in the ^1^H NMR spectrum, but reveals the presence of a singlet at δ_H_ 3.64 ppm, attributable to the −OCH_3_ group. Moreover, the ESI-MS spectrum of **6** recorded in CH_3_CN displays a negative ion at *m*/*z* 526, corresponding to [Ru_6_C(CO)_14_(COCH_3_)]^2–^ that results from monoelectron oxidation of **6** during ESI-MS analysis. The 2– charge of the ion in the mass spectrum is corroborated by a secondary peak at *m*/*z* 512, corresponding to the loss of a CO ligand from the oxidized **6**. Unfortunately, all attempts to crystallize **6** failed, hampering its complete structural characterization. Indeed, during crystallization, **6** partially decomposes and, among the decomposition products, it has been possible to identify **5,** as mentioned above. Thus, the structure of **6** has been computationally determined using DFT methods (Section 2.4). In all cases, the formulation of **6** must be done with care, since the present formulation is based solely on indirect and not very well resolved data.

The reactions of **2** in CH_3_CN with the stronger methylating agent CF_3_SO_3_CH_3_ resulted in mixtures of the new clusters [H_3_Ru_6_C(CO)_15_]^−^ (**7**) and [Ru_6_C(CO)_15_(CH_3_CNCH_3_)]^−^ (**8**), as well as the previously reported cluster **4**.^51^ All these species were structurally characterized by SC-XRD as their [NEt_4_][**4**], [NEt_4_][**7**] and [NEt_4_][**8**]**·**solv crystals.

**Figure 4:**
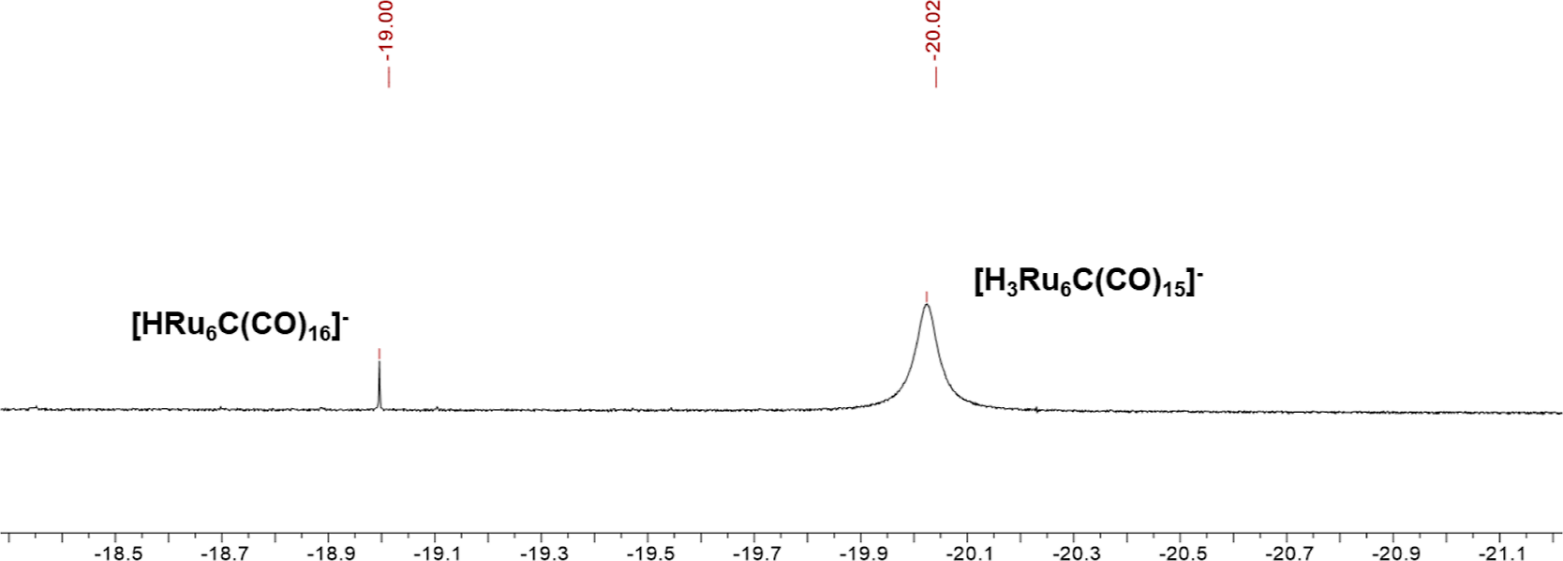
Hydride region of the ^1^H NMR spectrum of [H_3_Ru_6_(CO)_15_]^−^ (**7**) in CD_2_Cl_2_ at 298 K in the presence of minor traces of [HRu_6_(CO)_16_]^−^ (**4**).

[NEt_4_][**8**] was soluble in toluene and, thus, could be separated from [NEt_4_][**4**] and [NEt_4_][**7**], which were extracted in CH_2_Cl_2_. The two-hydride clusters could be distinguished by ^1^H NMR spectroscopy (Figures S12–S18 in the Supporting Information), since **4** displayed a sharp singlet at δ_H_ −19.00 ppm, whereas **7** showed a broader resonance at δ_H_ −20.02 ppm (Figure 4). In all cases, by carefully choosing the experimental conditions, it was possible to minimize the formation of **4**, resulting in almost pure **7** after work-up of the reaction mixture (see Section 4 for details, and Figures S6 and S9 in the Supporting Information for the IR spectra).

**Figure 5:**
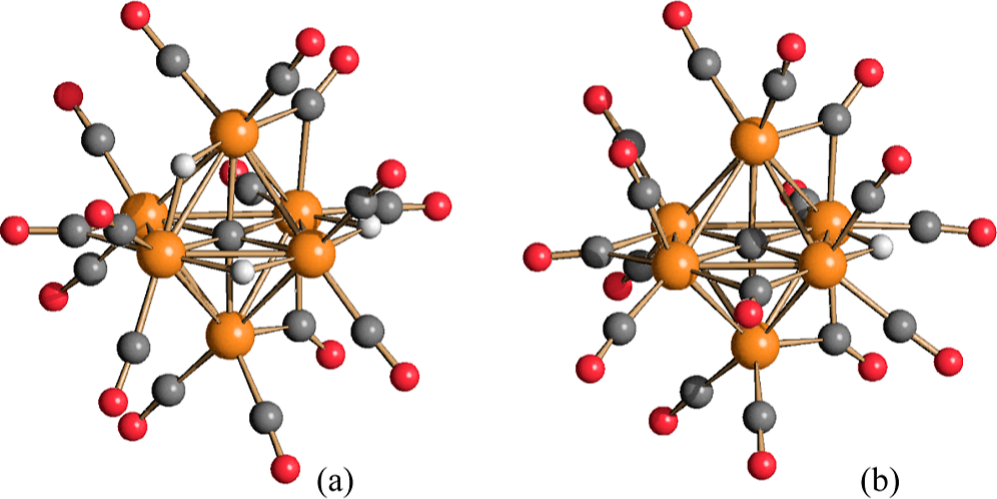
Molecular structures of (a) [H_3_Ru_6_C(CO)_15_]^−^ (**7**) and (b) [HRu_6_C(CO)_16_]^−^ (**4**) (orange Ru; red O; gray C; white H).

**Figure 6:**
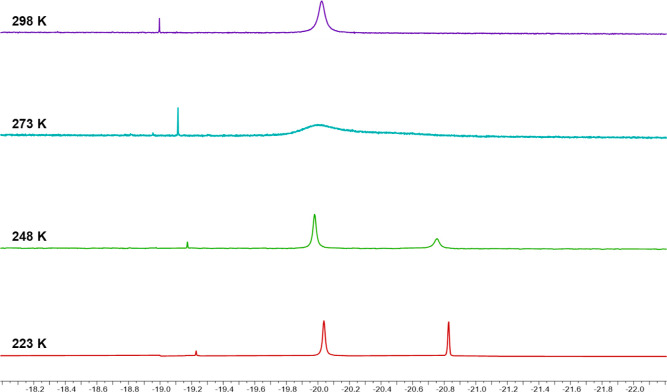
Hydride region of the VT ^1^H NMR spectra of [H_3_Ru_6_(CO)_15_]^−^ (**7**) in CD_2_Cl_2_. The sharp resonance at *ca.* δ_H_ −19.0 ppm is due to traces of **4**.

Cluster **4** was previously obtained from the reaction of **1** with acids.^51^ The structure of **4** was previously reported and, thus, it will be only briefly described herein and compared to **7**.^51^ They are both based on a common Ru_6_C-carbide-centered octahedral core with similar bonding parameters (Figure 5 and Table 1). There are 12 terminal carbonyls, 2 per each Ru atom, in both clusters and, in addition, **7** contains 3 μ-CO ligands, whereas 4 μ-CO ligands are present in **4**. The unique hydride of **4** is in an edge-bridging position. All the three hydride ligands of **7** are also edge bridging, two on the same face of the octahedron, whereas the third hydride is located on an adjacent edge. The trihydride nature of **7** was further corroborated by VT ^1^H NMR studies (Figure 6). Indeed, a broad resonance at δ_H_ −20.02 ppm was observed at 298 K, and coalescence occurred at 273 K. Eventually, two resonances at δ_H_ −20.04 (2H) and −20.83 (1H) ppm appeared at 223 K. The lower field resonance (2H) is broader than the higher field one (1H), suggesting that a further splitting could occur at even lower temperatures, in accordance with the SC-XRD structure where all three hydrides are not equivalent. Unfortunately, it was not possible to reach such a temperature.

The structure of **7** can be also compared to **3**. They both possess 12 terminal CO (2 per each Ru atom) and 3 edge-bridging CO, but their stereochemistry is slightly different (Figures 2 and 5), likely because of their different anionic charges and different number of hydride ligands. Thus, the three μ-CO ligands of trianionic **3** are located on three Ru–Ru edges without any Ru atom in common (not connected edges), whereas two μ-CO ligands of monoanionic **7** are located on two Ru–Ru edges with a common Ru atom, and the third μ-CO ligand on a not connected edge.

**Figure 7:**
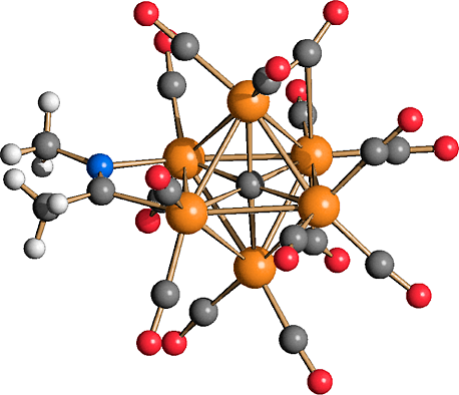
Molecular structure of [Ru_6_C(CO)_15_(CH_3_CNCH_3_)]^−^ (**8**) (orange Ru; red O; blue N; gray C; white H). Main bond distances (Å): Ru–Ru 2.831(2)–2.948(2), average 2.873(7); Ru–C_carbide_ 2.017(19)–2.042(18), average 2.03(4); Ru–N_imidoyl_ 2.067(18); Ru–C_imidoyl_ 2.07(2); C–N_imidoyl_ 1.23(3).

The molecular structure of **8** is based on an octahedral Ru_6_C core displaying one μ-CO ligand and one μ-imidoyl CH_3_CNCH_3_ ligand on two not-connected edges (Figure 7). The two Ru atoms not bonded to any edge bridging ligand bear three terminal carbonyls each, whereas the other four Ru atoms bear only two. The μ-imidoyl CH_3_CNCH_3_ ligand can be described as a three-electron donor if considered as a neutral ligand. Thus, overall **8** possesses 86 CVE as expected for an octahedron. The same μ-imidoyl CH_3_CNCH_3_ ligand was previously found in HOs_3_(CO)_10_(CH_3_CNCH_3_).^57^ The Ru–Ru and Ru–C_carbide_ bonding contacts are comparable to those found in other clusters containing the same Ru_6_C core.

### 2.3 Electrochemical and Spectroelectrochemical Studies of [Ru_6_C(CO)_15_]^4–^ (2) and [HRu_6_C(CO)_15_]^3–^ (**3**)

The electrochemical and IR spectroelectrochemical behavior of the carbide carbonyl clusters **1** and **2** and of the hydride **3** were revised/investigated in CH_3_CN/[NBu_4_][PF_6_] 0.1 M solution.

The electrochemical conversion of Ru_6_C(CO)_17_ into **1** and the back oxidation of **1**, in the presence of CO, to give Ru_6_C(CO)_17_ was previously reported.^58^ Controlled bulk electrolysis of [Ru_6_C(CO)_16_]^2–^ (**1**) performed under a steady stream of carbon monoxide was reported to consume 1.9 electrons and to produce Ru_6_C(CO)_17_ in near quantitative yield; on the other hand, the reduction of the neutral cluster at the suitable potential, under a constant stream of argon gas, was reported to consume 1.9 electrons and to give back **1** in quantitative yield.

We voltammetrically observed one irreversible reduction of **1** at the potential of −1.77 V *vs* Ag/AgCl and IR-SEC experiments confirmed the presence of the reduced tetraanion **2** [ν_CO_ 1890(vs) and 1708(m) cm^–1^] in the mixture of the reaction products (Figure S22 in the Supporting Information). The reduction of a CH_3_CN solution of **1** by controlled bulk electrolysis at a platinum gauze electrode at −1.8 V under a steady stream of argon required 1.73 mol of electrons per mol of cluster and an IR spectrum of the solution showed the presence of a mixture of products in which **2** constituted only a minor component, according to results of the spectroelecrochemical analysis.

**Figure 8:**
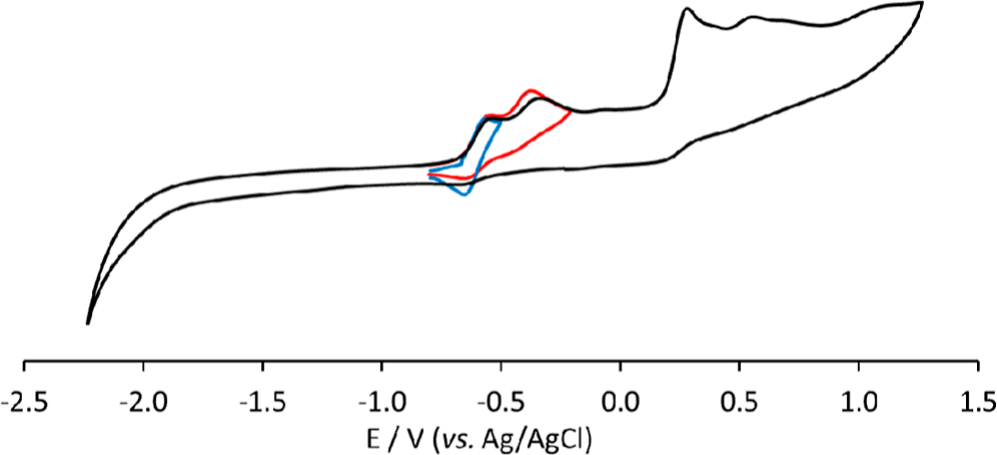
Cyclic voltammetry response of [Ru_6_C(CO)_15_]^4–^ (**2**) at a Pt electrode in CH_3_CN solution between −2.3 and +1.2 V, black line; between −0.8 and −0.2 V, red line; between −0.8 and −0.5 V blue line. [N^*n*^Bu_4_][PF_6_] (0.1 mol dm^–3^) supporting electrolyte, scan rate: 0.1 V s^–1^.

**Figure 9:**
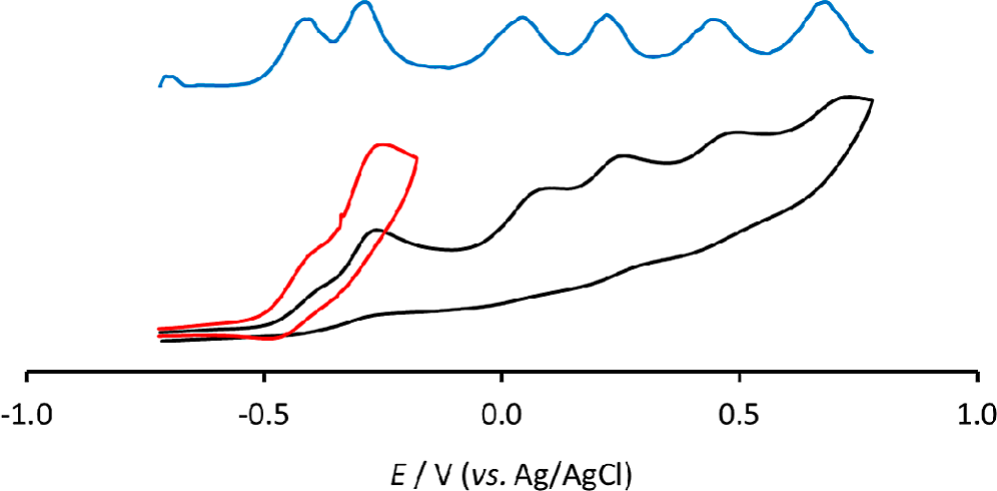
Differential pulse voltammetry (blue line) and cyclic voltammetry response of [HRu_6_C(CO)_15_]^3–^ (**3**) at a Pt electrode in CH_3_CN solution of [N^*n*^Bu_4_][PF_6_] (0.1 mol dm^–3^) supporting electrolyte: between −0.7 and +0.8 V (black line), scan rate: 0.1 V s^–1^; between −0.7 and −0.2 V (red line), scan rate: 0.2 V s^–1^.

The cyclovoltammetric profiles of the new clusters **2** and **3** in CH_3_CN/[NBu_4_][PF_6_] 0.1 M solution, under an inert atmosphere, are reported in Figures 8 and 9, respectively.

For the tetraanion **2**, one electrochemically and chemically reversible oxidation and one oxidation complicated by subsequent chemical reactions are observed at −0.60 and −0.42 V formal potentials, respectively, and these are followed by three irreversible processes at higher potentials (*E*_pa_ = +0.25, +0.52, and +0.83 V, respectively). A controlled potential coulometric measurement was performed at the potential *E*_w_ = −0.2 V in correspondence of both the first two very near oxidations of Figure 8 and proved that 1.8 electrons *per* mol of cluster were involved. An IR spectrum of the solution at the end of the electrolysis confirmed the quantitative formation of **5** (see also in situ IR SEC experiments).

Six oxidation processes characterize the cyclic voltammetry response of the hydride tri-anionic derivative **3**; relatively fast chemical reactions accompany all the electron removals. The first two oxidations recall in the shape those of the parent **2** and, accordingly to the decreased negative charge, are shifted toward more anodic potentials (−0.41 and −0.29 V); further oxidations occur at +0.04, +0.22, +0.46, and +0.68 V, respectively (half-wave potential values for all the processes obtained by the differential pulse voltammetry, DPV). By the comparison of the peak currents of cyclic voltammetries of **2** and **3**, we concluded that the same electron number, namely two, is involved in the first two unresolved oxidation redox steps.

The oxidation processes of **2** and **3** were investigated by in situ IR-SEC in an optically transparent thin-layer electrochemical (OTTLE) cell.^59^

**Figure 10:**
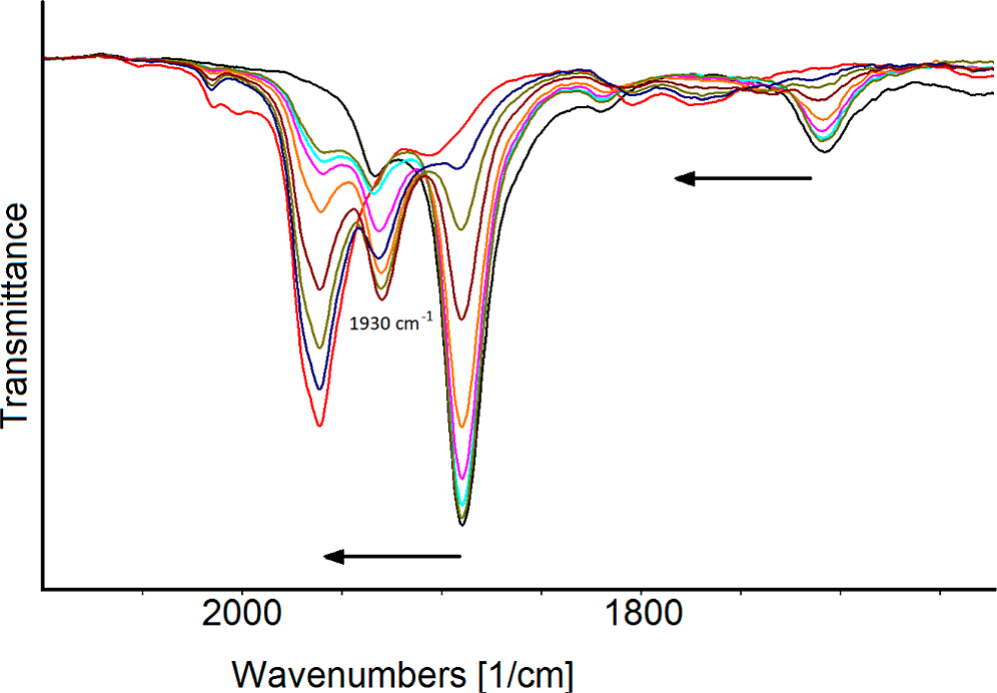
IR spectra of a CH_3_CN solution of [Ru_6_C(CO)_15_]^4–^ (**4**) recorded in an OTTLE cell during the progressive increase of the potential from −0.2 to +0.2 V (vs Ag pseudoreference electrode, scan rate 1 mV s^–1^). [N^*n*^Bu_4_][PF_6_] (0.1 mol dm^–3^) as the supporting electrolyte. The absorptions of the solvent and supporting electrolyte have been subtracted.

Figure 10 reports the IR spectra of **2** in CH_3_CN/[N^*n*^Bu_4_][PF_6_] solution recorded on increasing the working electrode (WE) potential from −0.2 to +0.2 V (vs Ag pseudo-reference electrode). The blue shift of the terminal and bridging CO stretching bands, from 1890 and 1708 cm^–1^ to 1963 and 1760 cm^–1^, points out the quantitative formation of the two-electrons oxidation product **5** isolated by the chemical oxidation of **2** in CH_3_CN and structurally characterized. The complete chemical reversibility of the electrochemical oxidation of **2** was ascertained by the restoration of its IR spectrum in the backward reduction step. Moreover, during the slow increase of the potential, in the early stage of the oxidation, two bands at 1930 and 1735 cm^–1^ increased their intensity up till a maximum and then decreased (Figure S23 in the Supporting Information). According to the cyclic voltammogram shown in Figure 8, where two close-spaced electron removal steps are evident, these absorptions can be tentatively attributed to the product of the one-electron oxidation of **2**.

**Figure 11:**
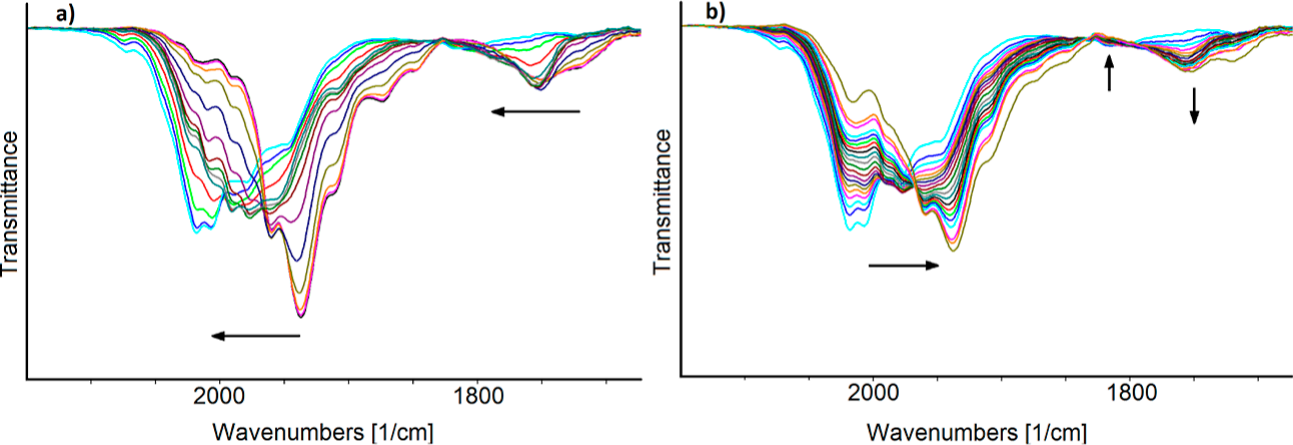
IR spectra of a solution of [HRu_6_C(CO)_15_]^3–^ (**3**) in CH_3_CN recorded in an OTTLE cell during (a) progressive increase of the WE potential from −0.1 to +0.7 V (vs Ag pseudoreference electrode; scan rate 1 mV s^–1^) and (b) during the reduction back-scan from +0.7 to −0.6 V (vs Ag pseudoreference electrode) [N^*n*^Bu_4_][PF_6_] (0.1 mol dm^–3^) as the supporting electrolyte. The absorptions of the solvent and supporting electrolyte have been subtracted.

The sequence of IR spectra of a solution of **3** in CH_3_CN/[N^*n*^Bu_4_][PF_6_] recorded in an OTTLE cell during the progressive increase of the WE potential from −0.1 to +0.7 V (vs Ag pseudo reference electrode) is reported in Figure 11a. A shift of the terminal and bridging CO bands at higher wavenumbers points out an oxidation of the hydride accompanied by a partial decomposition of the oxidized cluster and the initial spectrum was not completely restored in the reverse reduction scan (Figure 11b).

A thorough analysis of the IR spectra and the profile of the *i*/*E* curve made it possible to separate the complete sequence of the IR spectra in two groups, each belonging to a different redox step. The sequence of spectra showed in Figure S24a in the Supporting Information was collected by increasing the WE potential from −0.1 to + 0.4 V (vs Ag pseudoreference electrode). The IR spectrum of the starting cluster was re-obtained in the back-scan (Figure S24c in the Supporting Information) when the WE potential returned to a reducing value (Figure S24b in the Supporting Information), indicating a full chemical reversibility of the first redox process.

The spectra of **3** and of the products of its first and second oxidation are reported in Figure S25a in the Supporting Information. Upon oxidation, the profiles appear to be complicated by the presence of multiple overlapping bands, presumably due to deprotonation equilibria following the decrease of the negative charge in the CH_3_CN polar solvent. In an attempt to gain more information, we obtained the differential absorbance spectra (Figure S25b in the Supporting Information) where the initial spectrum of **3** (red line) and that of its first oxidation product (green line) are used to calculate, respectively, the difference spectra shown in black and red of Figure S25b in the Supporting Information. The absorbance maximum at 1980 cm^–1^ appears to be related to a monooxidation product of **3**, stable in the time scale of the spectroelectrochemical experiment, while the band at 2018 cm^–1^, that emerges in the difference spectrum after the second oxidation, can be tentatively attributed to a relatively instable monoanionic hydride that, as **5**, attains the 86 CVE through CH_3_CN coordination, and, eventually, to its decomposition products.

The electrochemistry of [Ru_6_C(CO)_17–*x*_]^2*x*−^ (*x* = 0–2) is dominated by the stability of the 86 CVE species, so the two-electron reduction of the neutral Ru_6_C(CO)_17_ is accompanied by the dissociation of a CO ligand to give **1**(58) that, in turn, can be further reduced losing CO to produce **2**. The reverse oxidation process of **1** yields a stable cluster only in the presence of CO whose coordination accompanies the two-electron removal. However, the oxidation of **2** in CH_3_CN solution appears to be a chemically reversible process in the absence of CO and the coordination of the solvent ensuring the attainment of 86 CVE. Moreover, bulk electrolysis and IR SEC experiments proved the electrochemical oxidation of **2** to be an improved synthesis of **5** with respect to the chemical one, typified by the absence of byproducts.

### 2.4 Computational Investigations

**Figure 12:**
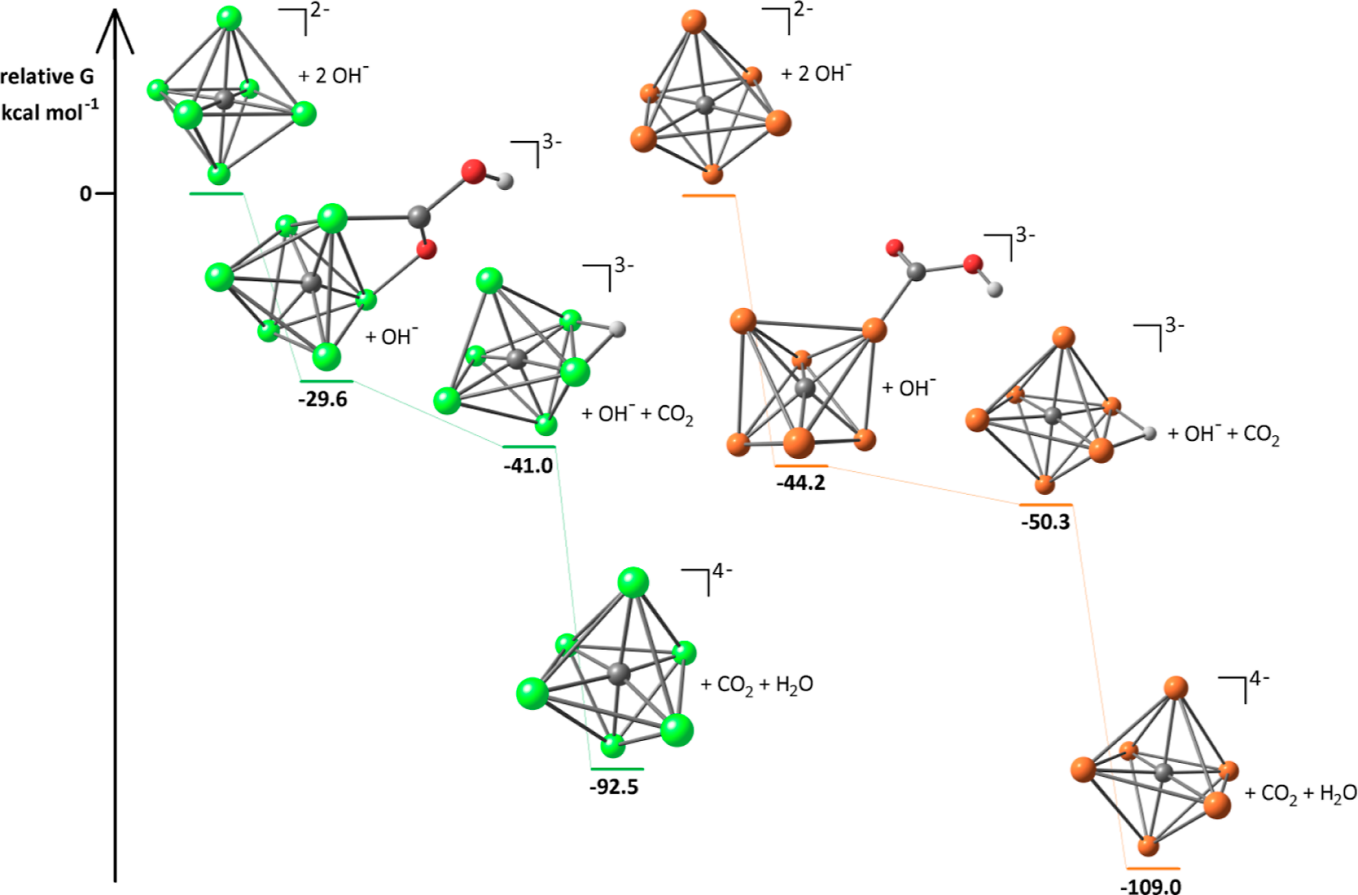
DFT-optimized geometries and relative Gibbs energy values of (from top to bottom) [M_6_C(CO)_16_]^2–^, [M_6_C(COOH)(CO)_15_]^3–^, [HM_6_C(CO)_15_]^3–^ and [M_6_C(CO)_15_]^4–^ (M = Fe, Ru). Color map: orange Ru; green Fe; red O; gray C; white H. Carbonyl ligands are omitted for clarity.

The DFT study on the ruthenium carbide carbonyl clusters, carried out at C-PCM/PBEh-3c level (DMSO as implicit solvent), studied the Gibbs energy variations related to the formation of **3** and **2** from **1** and OH^–^. For the sake of comparison, the calculations were also conducted on the analogous iron species at the same theoretical level.^56^ The relative Gibbs energy values are reported in Figure 12. The hydride position on a Ru–Ru edge in **3** was confirmed by the calculations (computed Ru–H distances 1.813 and 1.828 Å). The shift of the ν_CO_ stretching at lower wavenumbers moving toward more reduced species was confirmed by the unscaled simulated IR spectra and is shown in Figure S26 in the Supporting Information. For these compounds and the others computationally investigated (vide infra), the reduction of the computed wavenumbers by about 6% on applying suitable scaling factors allowed a good superposition with the experimental data.

The nucleophilic attack of OH^–^ on a carbonyl ligand, affording carboxylic complexes with general formula [M_6_C(COOH)(CO)_15_]^3–^, is associated to a meaningfully negative Gibbs energy variation, more pronounced for M = Ru: [Ru_6_C(CO)_16_]^2–^ + OH^–^ → [Ru_6_C(COOH)(CO)_15_]^3–^, Δ*G* = −44.2 kcal mol^–1^; [Fe_6_C(CO)_16_]^2–^ + OH^–^ → [Fe_6_C(COOH)(CO)_15_]^3–^, and Δ*G* = −29.6 kcal mol^–1^. It is worth noting that the predicted coordination mode of the formally anionic {COOH} ligand is different in the two clusters, that is, κ^1^-*C* for [Ru_6_C(COOH)(CO)_15_]^3–^ (computed Ru–C distance 2.058 Å) and μ-*C,O* for [Fe_6_C(COOH)(CO)_15_]^3–^ (computed Fe–C and Fe–O distances are 1.903 and 2.081 Å, respectively). The subsequent β-hydride elimination, affording [HM_6_C(CO)_15_]^3–^ and CO_2_, is another thermodynamically accessible reaction, in particular, for the iron derivative: [Ru_6_C(COOH)(CO)_15_]^3–^ → [HRu_6_C(CO)_15_]^3–^ + CO_2_, Δ*G* = −6.1 kcal mol^–1^; [Fe_6_C(COOH)(CO)_15_]^3–^ → [HFe_6_C(CO)_15_]^3–^ + CO_2_, Δ*G* = −11.4 kcal mol^–1^.

The deprotonation of the hydrides is more favorable for the ruthenium species, according to the Gibbs energy variations [HRu_6_C(CO)_15_]^3–^ + OH^–^ → [Ru_6_C(CO)_15_]^4–^ + H_2_O, Δ*G* = −58.7 kcal mol^–1^, and [HFe_6_C(CO)_15_]^3–^ + OH^–^ → [Fe_6_C(CO)_15_]^4–^ + H_2_O, Δ*G* = −51.5 kcal mol^–1^. On considering a weaker base such as NH_3_, the Δ*G* variations are 1.3 and 8.5 kcal mol^–1^ for M = Ru and M = Fe, respectively. [HFe_6_C(CO)_15_]^3–^ was only spectroscopically observed,^56^ and therefore, it is probably related to its faster decomposition with respect to the analogous ruthenium species and not to its acidity.

Computational studies were then addressed to selected products derived from the oxidation of **2**. The simple removal of two electrons should lead to the unsaturated species [Ru_6_C(CO)_15_]^2–^ (**9**), whose structure was simulated at C-PCM/PBEh-3c for the sake of clarity, despite the fact that only its CH_3_CN adduct **5** was experimentally isolated and structurally characterized. The DFT-optimized structure of **9** is comparable to that of **2** at the same theoretical level, the root-mean-square deviation (RMSD) between the two geometries being 0.453 Å. The similarity between the two clusters can be also observed from the superposition reported in Figure S27 in the Supporting Information. The simulated IR spectra of the two compounds, shown in Figure S27 in the Supporting Information, highlight the expected shift of the carbonyl stretching at higher wavenumbers for the less reduced **9**. The comparisons of the electron density (ρ) and potential energy density (V) values at Ru–carbide (3,–1) bond critical points (BCPs) indicate a slight weakening of the Ru–carbide bonds for the less reduced species. The Hirshfeld charges of the central cores are 0.187 and −0.097 a.u., respectively, for **9** and **2**; therefore, most of the charge variation between the two clusters is accounted by the carbonyl ligands. The different core charges are related to the ruthenium centers, while the partial charge of the central carbide remains almost constant (Table S1 in the Supporting Information).

The experimental observation of the CH_3_CN derivative **5** is justified by the very negative Gibbs energy variation for the reaction **9** + CH_3_CN → **5**, Δ*G* = −25.3 kcal mol^–1^. The DFT-optimized structure of **5** is in line with the X-ray outcomes, with RMSD value of 0.311 Å. The carbonyl regions of the unscaled simulated IR spectrum and the ground-state computed geometry are shown in Figure S28 in the Supporting Information. The computed ν_CO_ wavenumbers are quite similar to those calculated for **9**, despite the presence of the additional ligand CH_3_CN. Good agreement was observed from the comparison of the experimental and simulated IR spectra after proper scaling of the computed data (best scaling factor for **5** = 0.94).

As expected, CH_3_CN is a weaker ligand with respect to CO, given the Gibbs energy reaction for the reaction **5** + CO → **1** + CH_3_CN, −21.8 kcal mol^–1^.

Another cluster formally derived from the intermediate **9** is the trihydride [H_3_Ru_6_C(CO)_15_]^−^ (**7**) that can be considered as the product of the oxidative addition of H^+^ and H_2_. The thermodynamic of the two possible reactions involved appears to be characterized by negative Gibbs energy variations. On considering the ammonium ion as a model proton source, the reaction **9** + NH_4_^+^ → [HRu_6_C(CO)_15_]^−^ + NH_3_ has Δ*G* = −33.9 kcal mol^–1^. Such a reaction reveals the relatively basic character of **9**, since the computed Gibbs energy variation for the same reaction involving **2** and **3** is −1.3 kcal mol^–1^. The second formal step, [HRu_6_C(CO)_15_]^−^ + H_2_ → **7**, has Δ*G* = −23.5 kcal mol^–1^, the electronic saturation of the cluster probably being the driving force behind the process. The Cartesian coordinates of the supposed intermediate [HRu_6_C(CO)_15_]^−^ are provided for completeness as Supporting Information. The computed structure of **7** is in good agreement with the experimental data, the RMSD being 0.245 Å, and the position of the hydrides is confirmed. The simulated ν_CO_ stretching of **7** is in the same range of the monoanionic hydride **4**, while those of the trianion **3** fall at lower wavenumbers probably because of higher π-back-donation related to the whole negative charge of the cluster. The simulated IR spectra are superimposed in Figure S29 in the Supporting Information. It is worth noting that **7** is thermodynamically unstable in the presence of CO, since the reaction **7** + CO → **4** + H_2_ is associated to a Gibbs energy variation of −25.3 kcal mol^–1^.

The comparison of the AIM data related to the Ru–C_carbide_ bonds in the three hydrides indicates slightly weaker bonds for compound **7**, and the Hirshfeld population analysis revealed that the Ru centers are slightly more oxidized in this compound. The partial charge on the central carbide is instead almost the same in **3**, **4**, and **7**. The electron density required for the formation of the bonds with the three hydrogen atoms in **7** appears, therefore, almost in part drained from the metal centers and their bonds with the carbide. The average values of the Hirshfeld charges (Table S2) on the hydrogen atoms of **7** and **3** are quite similar (−0.102 and −0.110 a.u., respectively), whereas the charge on the hydride of **4** is slightly less negative (−0.087 a.u.). This is probably due to the presence of a further carbonyl ligand in **4** compared to **3** and **7**.

Another product of the reaction between **2** and CF_3_SO_3_CH_3_ is the cluster **8**, with the [CH_3_CNCH_3_]^+^ ligand in the coordination sphere. The DFT-optimized structure is in line with the X-ray outcomes, having an RMSD value of 0.310 Å. The carbonyl region of the simulated IR spectrum and the ground-state computed geometry are shown in Figure S30 in the Supporting Information. The less negative charge of **8** with respect to **5** probably accounts for the slight increase of computed terminal ν_CO_ frequencies (compare Figure S30 and S28 in the Supporting Information). The unscaled ν_CN_ stretching is predicted (with low intensity) at 1757 cm^–1^. For comparison, the calculated ν_CN_ stretching of **5** is 2490 cm^–1^, in line with the change of CN bond order. The Hirshfeld charges of the {Ru_6_C} core in **8** is 0.309 a.u., meaningfully more positive with respect to **5**, which is 0.178 a.u., an effect attributable to the coordination of a formally positively charged ligand in **8** (Table S3 in the Supporting Information). Charge decomposition analysis (CDA) calculations on **8** revealed a noticeable Lewis acidity of [CH_3_CNCH_3_]^+^. The computed donation from the ligand to the ruthenium carbide carbonyl fragment is 0.189 electrons, while the reverse process accounts for 0.169 electrons. According to the AIM data collected in Table S3 in the Supporting Information, the Ru–C(carbide) interactions are stronger in **8** with respect to **5**.

**Figure 13:**
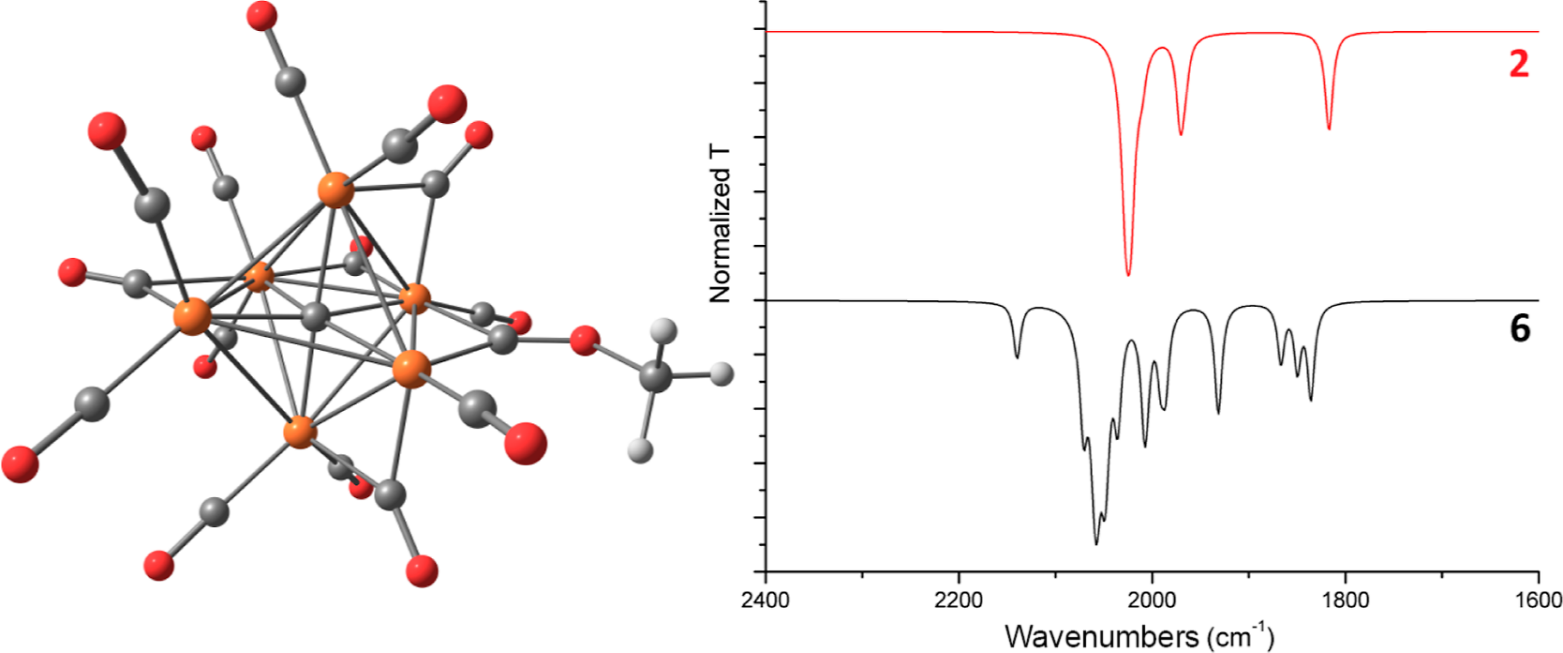
DFT-optimized structure of [Ru_6_C(CO)_14_(COCH_3_)]^3–^ (**6**) and simulated IR spectra of **6** and **2** (Lorentzian-broadening functions, fwhm = 8 cm^–1^). Color map: orange Ru; red O; gray C; white H.

The reaction of **2** with CH_3_I led to the isolation of the methylated species [Ru_6_C(CO)_14_(COCH_3_)]^3–^ (**6**). Several attempts to optimize the geometry starting from that of **2** with the addition of a [CH_3_]^+^ fragment on the oxygen atoms were carried out, and the lowest energy stationary point found is depicted in Figure 13. The carbyne ligand formed by the methylation of CO asymmetrically bridges two Ru centers with computed Ru–C bond lengths of 1.845 and 2.055 Å. The asymmetry of the bonds is also evidenced by the computed values at the respective (3,–1) BCPs summarized in Table 2. The unscaled carbonyl region of the simulated IR spectrum is slightly shifted toward higher wavenumbers with respect to the parent cluster **2** (Figure 13; best scaling factor for **2** and **6** = 0.94), suggesting that the {Ru_6_C} core is less electron-rich. The Hirshfeld charge of the {Ru_6_C} core is in fact 0.035 a.u. (carbide charge −0.385 a.u.; Ru average charge 0.070 a.u.), while the calculated value for **2** is −0.097 a.u. Despite the formal attack of [CH_3_]^+^ on a CO ligand, the partial charge of the {COCH_3_} fragment is close to zero (0.001 a.u.). Concerning the Ru–C(carbide) bonds, the comparison of the AIM data collected in Table 2 with those in Table S1 in the Supporting Information indicates a slight weakening with respect to compound **2**.

**Table 2:** AIM Data for the Ru–Carbide and Ru–Carbyne Bonds of [Ru_6_C(CO)_14_(COCH_3_)]^3–^ (**6**)

bond	ρ (e Å^–3^)	*V* (hartree Å^–3^)	*E* (hartree Å^–3^)	∇2ρ (e Å^–5^)
Ru–C(carbyne), short	1.258	–1.948	–0.751	6.365
Ru–C(carbyne), long	0.821	–1.044	–0.338	5.265
Ru–C(carbide), average	0.803	–1.061	–0.346	5.279

The C(carbyne)-O stretching is combined with C–H bending vibrations, and it is thus related to two bands simulated at 1379 and 1543 cm^–1^.

## 3 Conclusions

The reduction of **1** afforded the highly reduced tetraanion **2** that represented an interesting platform for the synthesis of further Ru–carbide carbonyl clusters. Indeed, protonation of **2** resulted in the monohydride **3**, whereas oxidation of **2** under different experimental conditions allowed the synthesis of **4–8**. Clusters **1** and **4** were previously described,^50−55^ whereas **2**, **3**, **5**, **6**, **7,** and **8** were reported here for the first time. Clusters **1–8** are all based on the same octahedral Ru_6_C core and display 86 CVE. Based on the present findings, it is likely that the previously reported unsaturated 84 CVE cluster [HRu_6_C(CO)_15_]^−^^53^ could be better reformulated as the trihydride **7**. Indeed, they show almost identical structures and, in addition, the purported [HRu_6_C(CO)_15_]^−^ was obtained under H_2_ pressure. It must be remarked that **7** could not be obtained by further protonation of **3**, since the latter species decomposed upon addition of acids. Thus, **7** could only be obtained upon treatment of **2** with CF_3_SO_3_CH_3_ under controlled experimental conditions.

It is noteworthy that two-electron oxidation of **2**, by chemical and electrochemical methods afforded the dianion **5** upon coordination of a CH_3_CN molecule in order to maintain 86 CVE. The fact that such Ru–carbide carbonyl clusters can undergo redox reactions as well as protonation/deprotonation reactions, resulting in hydride species, might be of interest for electrocatalysis, as recently found for Fe and Co carbide carbonyl clusters.^25−29^

Overall, the Ru_6_C framework appears to be very robust, may exist with charges ranging from 0 to −4, and can coordinate several different combinations of ligands on its surface, allowing for the isolation of a large variety of clusters.

## 4 Experimental Section

### 4.1 General Procedures

All reactions and sample manipulations were carried out using standard Schlenk techniques under nitrogen and in dried solvents. All the reagents were commercial products (Sigma-Aldrich) of the highest purity available and used as received, except [NEt_4_][HRu_3_(CO)_11_], which has been prepared according to the literature.^60^ Analyses of C, H, and N were carried out with a Thermo Quest Flash EA 1112NC instrument. IR spectra were recorded on a PerkinElmer Spectrum One interferometer in CaF_2_ cells. ^1^H NMR measurements were performed on a Varian Mercury Plus 400 MHz instrument. The proton chemical shifts were referenced to the nondeuterated aliquot of the solvent. Structure drawings have been performed with SCHAKAL99.^61^

### 4.2 Synthesis of [NEt_4_]_4_[Ru_6_C(CO)_15_] ([NEt_4_]_4_[2]) in Mixture with [NEt_4_]_3_[HRu_6_C(CO)_15_] ([NEt_4_]_3_[3])


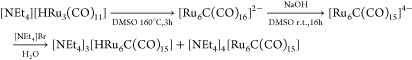
A solution of [NEt_4_][HRu_3_(CO)_11_] (0.500 g, 0.674 mmol) in 10 mL of DMSO was heated at 160 °C for 3 h, and the reaction was monitored by IR spectroscopy until the quantitative formation of **1**. After cooling down the solution to room temperature, NaOH (1.20 g) was added as a solid and, then, the reaction mixture was stirred for 16 h at room temperature. After removal of NaOH pellets, the crude product was precipitated by addition of a saturated solution of [NEt_4_]Br in H_2_O (100 mL). The resulting solid was recovered by filtration, washed with H_2_O (3 × 15 mL), toluene (15 mL), and THF (15 mL), and extracted with acetone (15 mL). Crystals of [NEt_4_]_3_[HRu_6_C(CO)_15_] ([NEt_4_]_3_[**3**]) suitable for X-ray analyses were obtained by layering *n*-hexane (30 mL) on the acetone solution (yield 43%). Then, the residue was extracted in acetonitrile and layered with *n*-hexane (2 mL) and diisopropyl ether (30 mL) affording crystals of [NEt_4_]_4_[Ru_6_C(CO)_15_]**·**CH_3_CN ([NEt_4_]_4_[**2**]**·**CH_3_CN) suitable for X-ray analysis (yield 37%).

[NEt_4_]_3_[**3**]: C_40_H_61_N_3_O_15_Ru_6_ (1430.33): calcd. (%): C, 33.59; H, 4.30; N, 2.94. Found: C, 33.82; H, 4.01; N, 3.18. IR (CH_3_CN, 298 K) ν_CO_: 1935(s), 1746(m) cm^–1^. ^1^H NMR (CD_3_CN, 298 K): δ −19.18 ppm.

[NEt_4_]_4_[**2**]**·**CH_3_CN: C_50_H_83_N_5_O_15_Ru_6_ (1600.63): calcd. (%): C, 37.52; H, 5.23; N, 4.38. Found: C, 37.23; H, 5.39; N, 4.05. IR (CH_3_CN, 298 K) ν_CO_: 1890(vs), 1708(m) cm^–1^. IR (Nujol, 298 K) ν_CO_: 1934(m), 1860(s) (m), 1744(w) cm^–1^.

### 4.3 Optimized Synthesis of [NEt_4_]_4_[Ru_6_C(CO)_15_] ([NEt_4_]_4_[**2**])


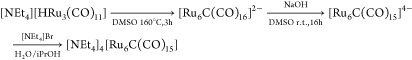
A solution of [NEt_4_][HRu_3_(CO)_11_] (0.700 g, 0.943 mmol) in 15 mL of DMSO was heated at 150 °C for 3 h, and the reaction was monitored by IR spectroscopy until quantitative formation of **1**. The solution was cooled down to room temperature, and powdered NaOH (1.70 g) was added. Then, the suspension was stirred for 16 h at room temperature. At the end of the reaction, the solution, decanted from NaOH powder, was added dropwise to a stirred solution of [NEt_4_]Br (3.00 g) in H_2_O (15 mL) and ^i^PrOH (100 mL). The solid was recovered upon filtration, washed with H_2_O (2 × 20 mL), and dried in vacuum. The solid was then extracted in CH_3_CN (15 mL) and layered with *n*-hexane (2 mL) and diisopropyl ether (30 mL) affording crystals of [NEt_4_]_4_[Ru_6_C(CO)_15_]**·**CH_3_CN ([NEt_4_]_4_[**2**]**·**CH_3_CN) suitable for X-ray analysis (yield 85%).

[NEt_4_]_4_[**2**]**·**CH_3_CN: C_50_H_83_N_5_O_15_Ru_6_ (1600.63): calcd. (%): C, 37.52; H, 5.23; N, 4.38. Found: C, 37.23; H, 5.39; N, 4.05. IR (CH_3_CN, 298 K) ν_CO_: 1890(vs), 1708(m) cm^–1^. IR (Nujol, 298 K) ν_CO_: 1934(m), 1860(s) (m), 1744(w) cm^–1^.

### 4.4 Synthesis of [NEt_4_]_3_[HRu_6_C(CO)_15_] ([NEt_4_]_3_[**3**])


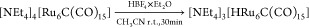
[NEt_4_]_4_[**2**] (0.250 g, 0.160 mmol) was dissolved in CH_3_CN (15 mL), and a solution of HBF_4_**·**Et_2_O (22 μL, 0.160 mmol) in CH_3_CN (2 mL) was added dropwise. The reaction mixture was stirred at room temperature and was monitored by IR spectroscopy. At the end of the reaction, the solvent was removed in vacuum and the residue was washed with water (2 × 20 mL) and toluene (10 mL). Then, the solid was dried under reduced pressure and extracted with CH_2_Cl_2_ (10 mL). Crystals of [NEt_4_]_3_[HRu_6_C(CO)_15_] ([NEt_4_]_3_[**3**]) suitable for X-ray analyses were obtained by slow diffusion of *n*-pentane (20 mL) on the CH_2_Cl_2_ solution (yield 72%).

[NEt_4_]_3_[**3**]: C_40_H_61_N_3_O_15_Ru_6_ (1430.33): calcd. (%): C, 33.59; H, 4.30; N, 2.94. Found: C, 33.82; H, 4.01; N, 3.18. IR (CH_3_CN, 298 K) ν_CO_: 1935(s), 1746(m) cm^–1^. ^1^H NMR (CD_3_CN, 298 K): δ −19.18 ppm.

### 4.5 Synthesis of [NEt_4_]_2_[Ru_6_C(CO)_15_(CH_3_CN)] ([NEt_4_]_2_[**5**])

#### 4.5.1 From [NEt_4_]_4_[Ru_6_C(CO)_15_] ([NEt_4_]_4_[**2**]) and [Cp_2_Fe][PF_6_]


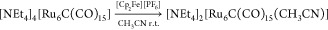
[Cp_2_Fe][PF_6_] (0.127 g, 0.385 mmol) in CH_3_CN (2 mL) was added dropwise to a solution of [NEt_4_]_4_[**2**] (0.300 g, 0.192 mmol) in CH_3_CN (20 mL). The reaction mixture was stirred at room temperature and was monitored by IR spectroscopy. The reaction was considered concluded when the ν_CO_ band of **2** disappeared and was replaced by a ν_CO_ band at 1963 cm^–1^. At the end of the reaction, the solvent was removed in vacuum and the residue was washed with water (2 × 20 mL) and toluene (10 mL). The solid was dried under reduced pressure and extracted with CH_2_Cl_2_ (15 mL). After filtration, the solvent was removed in vacuum affording a microcrystalline solid of [NEt_4_]_2_[Ru_6_C(CO)_15_(CH_3_CN)] ([NEt_4_]_2_[**5**]) (yield 61%). This compound was identified by IR spectroscopy comparing its spectrum with that of the crystals of [NEt_4_]_2_[**5**] obtained in Section 4.6.

[NEt_4_]_2_[Ru_6_C(CO)_15_(CH_3_CN)]: C_34_H_43_N_3_O_15_Ru_6_ (1340.13): calcd. (%): C, 30.47; H, 3.23; N, 3.14. Found: C, 30.11; H, 3.07; N, 3.31. IR (CH_3_CN, 298 K) ν_CO_: 1963(vs), 1760(m) cm^–1^. ^1^H NMR (CD_3_CN, 298 K): δ 2.60 (CH_3_CN) ppm.

#### 4.5.2 From [NEt_4_]_4_[Ru_6_C(CO)_15_] ([NEt_4_]_4_[**2**]) and [C_7_H_7_][BF_4_]


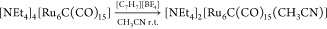
[C_7_H_7_][BF_4_] (0.028 g, 0.158 mmol) in CH_3_CN (2 mL) was added dropwise to a solution of [NEt_4_]_4_[**2**] (0.250 g, 0.158 mmol) in CH_3_CN (15 mL). The reaction mixture was stirred at room temperature and was monitored by IR spectroscopy. The reaction was considered concluded when the ν_CO_ band of **2** disappeared and was replaced by a ν_CO_ band at 1963 cm^–1^. At the end of the reaction, the solvent was removed in vacuum and the residue was washed with water (2 × 20 mL) and toluene (10 mL). The solid was dried under reduced pressure and extracted with CH_2_Cl_2_ (15 mL). The presence of **5** in solution was confirmed by IR spectroscopy. This reaction demonstrated to be less selective compared to those performed with [Cp_2_Fe][PF_6_] due to the formation of side products such as **1** (yield 36%).

### 4.6 Reaction of [NEt_4_]_4_[Ru_6_C(CO)_15_] ([NEt_4_]_4_[**2**]) with CH_3_I: Synthesis of [NEt_4_]_3_[Ru_6_C(CO)_14_(COCH_3_)] ([NEt_4_]_3_[**6**]) and Crystal Structure of [NEt_4_]_2_[Ru_6_C(CO)_15_(CH_3_CN)] ([NEt_4_]_2_[**5**])

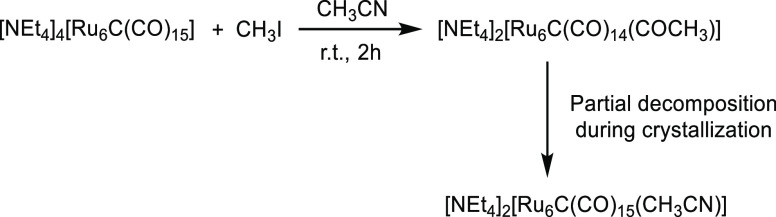



CH_3_I (0.048 mL, 0.770 mmol) in CH_3_CN (2 mL) was added dropwise to a solution of [NEt_4_]_4_[**2**] (0.300 g, 0.192 mmol) in CH_3_CN (20 mL). The mixture was stirred at room temperature for 2 h and monitored by IR spectroscopy, and the solvent was removed in vacuum. The residue was washed with water (2 × 20 mL), toluene (10 mL), and CH_2_Cl_2_ (10 mL) and was extracted with CH_3_CN (15 mL). IR and ESI-MS analyses indicated that the major product present in solution was **6**. Nonetheless, **6** partially decomposed during the diffusion of *n*-hexane and diisopropyl ether on CH_3_CN solution affording a few crystals of [NEt_4_]_2_[Ru_6_C(CO)_15_(CH_3_CN)] ([NEt_4_]_2_[**5**]) suitable for X-ray analyses (yield 17%).

[NEt_4_]_3_[**6**]: IR (CH_3_CN, 298 K) ν_CO_: 1939(vs), 1748 cm^–1^. ESI-MS (*m*/*z*): ES– 526 [M]^2–^, 512 [M – CO]^2–^; ES+ 130 [NEt_4_]^+^. ^1^H NMR (CD_3_CN, 298 K): δ 3.64 (OCH_3_) ppm.

[NEt_4_]_2_[**5**]: IR (CH_3_CN, 298 K) ν_CO_: 1963(vs), 1760(m) cm^–1^.

### 4.7 Synthesis of [NEt_4_][H_3_Ru_6_C(CO)_15_] ([NEt_4_][**7**])


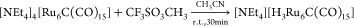
CF_3_SO_3_CH_3_ (22 μL, 0.199 mmol) was added to a solution of [NEt_4_]_4_[**2**] (0.310 g, 0.199 mmol) in CH_3_CN (20 mL). The mixture was stirred at room temperature for 30 min and, the solvent was removed in vacuum. The residue was washed with water (2 × 20 mL) and toluene (10 mL), dried under reduced pressure, and then extracted with CH_2_Cl_2_ (10 mL). Crystals of [NEt_4_][H_3_Ru_6_C(CO)_15_] ([NEt_4_][**7**]) suitable for X-ray analyses were obtained by slow diffusion of *n*-pentane (20 mL) on the CH_2_Cl_2_ solution (yield 67%). The hydrogen atoms of **7** probably originate from traces of water present in the reaction medium.

C_24_H_23_NO_15_Ru_6_ (1171.85): calcd. (%): C, 24.60; H, 1.98; N, 1.96. Found: C, 24.28; H, 2.040; N, 1.55. IR (CH_2_Cl_2_, 298 K) ν_CO_: 2016(vs), 1821(m) cm^–1^. IR (Nujol, 298 K) ν_CO_: 2000(s), 1770(m) cm^–1^. ^1^H NMR (CD_2_Cl_2_ 298 K): δ −20.02 (br) ppm; ^1^H NMR (CD_2_Cl_2_ 223 K): δ −20.04 (2H), −20.83 (1H) ppm.

### 4.8 Synthesis of [NEt_4_][HRu_6_C(CO)_16_] ([NEt_4_][**4**]) and [NEt_4_][Ru_6_C(CO)_15_(CH_3_CNCH_3_)]·solv (NEt_4_][**8**]·solv)


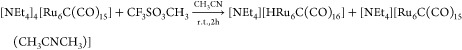
CF_3_SO_3_CH_3_ (76 μL 0.693 mmol) was added dropwise to a solution of [NEt_4_]_4_[**2**] (0.360 g, 0.231 mmol) in CH_3_CN (20 mL). The mixture was stirred at room temperature for 2 h, and then the solvent was removed in vacuum. The residue was washed with water (2 × 20 mL), dried under reduced pressure, and extracted with toluene (10 mL). Crystals of the [NEt_4_][Ru_6_C(CO)_15_(CH_3_CNCH_3_)]**·**solvent ([NEt_4_][**8**]**·**solv) suitable for X-ray analyses were obtained by slow diffusion of *n-*pentane (20 mL) on the toluene solution (yield 21%). Then, the residue, not soluble in toluene, was extracted in CH_2_Cl_2_ (10 mL) and layered with *n-*pentane (20 mL) affording crystals of [NEt_4_][HRu_6_C(CO)_16_] ([NEt_4_][**4**]) suitable for X-ray analysis (yield 46%).

[NEt_4_][**8**]**·**solv: IR (CH_2_Cl_2_, 298 K) ν_CO_: 2052(w), 2001(s) cm^–1^. IR (Nujol, 298 K) ν_CO_: 2054(w), 1978(s) cm^–1^. ^1^H NMR (CD_2_Cl_2_, 298 K): δ 2.60 (CH_3_CN), 3.41 (CNCH_3_) ppm.

[NEt_4_][**4**]: IR (CH_2_Cl_2_, 298 K) ν_CO_: 2011(vs), 1811(m) cm^–1^. IR (Nujol, 298 K) ν_CO_: 2002 (s), 1811 (m) cm^–1^. ^1^H NMR (CD_2_Cl_2_, 298 K): δ −18.98 (br) ppm.

### 4.9 X-Ray Crystallographic Study

Crystal data and collection details for [NEt_4_]_4_[**2**]**·**CH_3_CN, [NEt_4_]_3_[**3**], [NEt_4_][**7**], [NEt_4_][**4**], [NEt_4_][**8**]**·**solv, [NEt_4_]_2_[**5**], [NEt_4_][RuCl_3_(CO)_2_(CH_3_CN)_2_] are reported in Table S4 in the Supporting Information. The diffraction experiments were carried out on a Bruker APEX II diffractometer equipped with a PHOTON2 detector using Mo–Kα radiation. Data were corrected for Lorentz polarization and absorption effects (empirical absorption correction SADABS).^62^ Structures were solved by direct methods and refined by full-matrix least-squares based on all data using *F*^2^.^63^ Hydrogen atoms were fixed at calculated positions and refined by a riding model. All non-hydrogen atoms were refined with anisotropic displacement parameters, unless otherwise stated. The crystals of [NEt_4_]_3_[**3**] appeared to be non-merohedrally twinned. The TwinRotMat routine of PLATON was used to determine the twinning matrix and to write the reflection data file (.hkl) containing the twin components.^64^ The unit cell of [NEt_4_][**8**]**·**solv contains an additional total potential solvent accessible void of 947 Å^3^ (ca. 12% of the cell volume), which is likely to be occupied by highly disordered solvent molecules. These voids have been treated using the SQUEEZE routine of PLATON.^65^

### 4.10 Electrochemical and Spectroelectrochemical Measurements

Electrochemical measurements were performed with a PalmSens4 instrument interfaced to a computer employing PSTrace5 electrochemical software. Cyclic voltammetry measurements were carried out at room temperature under argon in CH_3_CN solutions containing [N^*n*^Bu_4_][PF_6_] (0.1 mol dm^–3^) as the supporting electrolyte. HPLC grade CH_3_CN (Sigma-Aldrich) was stored under argon over 3 Å molecular sieves. Electrochemical grade [N^*n*^Bu_4_][PF_6_] was purchased from Fluka and was used without further purification. Cyclic voltammetry was performed in a three-electrode cell, and the working and the counterelectrodes consisted of a Pt disk and a Pt gauze, respectively, both sealed in a glass tube. A leakless miniature Ag/AgCl/KCl electrode (eDAQ) was employed as a reference. The three-electrode home-built cell was predried by heating under vacuum and filled with argon. The Schlenk-type construction of the cell maintained anhydrous and anaerobic conditions. The solution of supporting electrolyte, prepared under argon, was introduced into the cell and the cyclic voltammetry of the solvent was recorded. The analyte was then introduced and voltammograms were recorded. Under the present experimental conditions, the one-electron oxidation of ferrocene occurs at *E*° = +0.42 V vs Ag/AgCl.

Controlled potential coulometry was performed in an H-shaped cell with anodic and cathodic compartments separated by a sintered-glass disk. The working macroelectrode and counterelectrode were platinum gauze. CH_3_CN presaturated argon was bubbled in the solution during the reduction of **1**.

Infrared (IR) spectroelectrochemical measurements were carried out using an optically transparent thin-layer electrochemical (OTTLE) cell^59^ equipped with CaF_2_ windows, platinum mini-grid working and auxiliary electrodes and silver wire pseudoreference electrode. During the microelectrolysis procedures, the electrode potential was controlled by a PalmSens4 instrument interfaced to a computer employing PSTrace5 electrochemical software. Argon-saturated CH_3_CN solutions of the compound under study, containing [N^*n*^Bu_4_][PF_6_] 0.1 M as the supporting electrolyte, were used. The in situ spectroelectrochemical experiments have been performed by collecting spectra of the solution at constant time intervals during the oxidation or reduction obtained by continuously increasing or lowering the initial working potential at a scan rate of 1.0 mV/s. IR spectra were recorded on a PerkinElmer Spectrum 100 FT-IR spectrophotometer.

### 4.11 Computational Details

Geometry optimizations were performed using the PBEh-3c method, which is a reparametrized version of PBE0^66,67^ (with 42% HF exchange) that uses a split-valence double-zeta basis set (def2-mSVP)^68,69^ and adds three corrections considering dispersion, basis set superposition, and other basis set incompleteness effects.^70−72^ The C-PCM implicit solvation model was added to DFT calculations, considering a dielectric constant of 46.68 and a refractive index of 1.4793.^73,74^ IR simulations were carried out using the harmonic approximation, from which zero-point vibrational energies and thermal corrections (*T* = 298.15 K) were obtained.^75^ The software used was ORCA version 5.0.3.^76^ The output was elaborated using MultiWFN, version 3.8.^77^ Cartesian coordinates of the DFT-optimized structures are collected in a separate .xyz file.

## Supporting Information Available

Experimental and computational information, crystal data, and collection details (PDF)
DFT-optimized coordinates (XYZ)

## Supplementary Material

ic3c01711_si_001.pdf

ic3c01711_si_002.xyz

## References

[ref1] Johnson B. F. G.; Jonston R. D.; Lewis J. Ruthenium carbonyl carbide compounds. Chem. Commun. 1967, 1057 10.1039/c1967001057a.

[ref2] Sirigu A.; Bianchi M.; Benedetti E. The Crystal Structure of Ru_6_C(CO)_17_. Chem. Commun. 1969, 596 10.1039/c2969000596a.

[ref3] Eady C. R.; Johnson B. F. G.; Lewis J. The Chemistry of Polynuclear Compounds. Part XXVI. Products of the Pyrolysis of Dodecacarbonyl-triangulo-triruthenium and -triosmium. J. Chem. Soc., Dalton Trans. 1975, 2606–2611. 10.1039/dt9750002606.

[ref4] Cesari C.; Femoni C.; Carmela Iapalucci M.; Zacchini S. Molecular Fe, Co and Ni carbide carbonyl clusters and nanoclusters. Inorg. Chim. Acta 2023, 544, 121235 10.1016/j.ica.2022.121235.

[ref5] Reinholdt A.; Bendix J. Transition Metal Carbide Complexes. Chem. Rev. 2022, 122, 830–902. 10.1021/acs.chemrev.1c00404. 34797626

[ref6] Takemoto S.; Matsuzaka H. Recent advances in the chemistry of ruthenium carbido complexes. Coord. Chem. Rev. 2012, 256, 574–588. 10.1016/j.ccr.2011.10.025.

[ref7] Metal Clusters in Chemistry; Braunstein P., Oro L. A., Raithby P. R., Eds.; Wiley VCH: Weinheim, 1999.

[ref8] Cesari C.; Shon J.-H.; Zacchini S.; Berben L. A. Metal carbonyl clusters of groups 8-10: synthesis and catalysis. Chem. Soc. Rev. 2021, 50, 9503–9539. 10.1039/d1cs00161b. 34259674

[ref9] Barik C. K.; Ganguly R.; Li Y.; Samanta S.; Leong W. K. Reaction of the Decaosmium Carbido Cluster [Os_10_(μ_6_-C)(CO)_24_]^2–^ with Halostibines. J. Cluster Sci. 2021, 32, 929–935. 10.1007/s10876-020-01857-w.

[ref10] Adams R. D.; Akter H.; Kaushal M.; Smith M. D.; Tedder J. D. Synthesis, Structures, and Transformations of Bridging and Terminally-Coordinated Trimethylammonioalkenyl Ligands in Zwitterionic Pentaruthenium Carbido Carbonyl Complexes. Inorg. Chem. 2021, 60, 3781–3793. 10.1021/acs.inorgchem.0c03541. 33616385

[ref11] Adams R. D.; Smith M. D.; Wakdikar N. D. Zwitterionic Ammoniumalkenyl Ligands in metal Cluster Complexes. Synthesis, Structures, and Transformations of Zwitterionic Trimethylammoniumalkenyl Ligands in Hexaruthenium Carbido Carbonyl Complexes. Inorg. Chem. 2020, 59, 1513–1521. 10.1021/acs.inorgchem.9b03349. 31885256

[ref12] Johnson B. F. G.; Martin C. M. The Role of Interstitial Atoms in Transition Metal Carbonyl Clusters. Metal Clusters in Chemistry; Braunstein P., Oro L. A., Raithby P. R., Eds.; Wiley VCH: Weinheim, 1999; pp 877–912.

[ref13] Femoni C.; Iapalucci M. C.; Kaswalder F.; Longoni G.; Zacchini S. The possible role of metal carbonyl clusters in nanoscience and nanotechnologies. Coord. Chem. Rev. 2006, 250, 1580–1604. 10.1016/j.ccr.2006.03.011.

[ref14] Bernardi A.; Ciabatti I.; Femoni C.; Iapalucci M. C.; Longoni G.; Zacchini S. Molecular nickel poly-carbide carbonyl nanoclusters: The octa-carbide [HNi_42_C_8_(CO)_44_(CuCl)]^7–^ and the deca-carbide [Ni_45_C_10_(CO)_46_]^6–^. J. Organomet. Chem. 2016, 812, 229–239. 10.1016/j.jorganchem.2015.09.013.

[ref15] Capacci C.; Cesari C.; Femoni C.; Iapalucci M. C.; Mancini F.; Ruggieri S.; Zacchini S. Structural Diversity in Molecular Nickel Phosphide Carbonyl Nanoclusters. Inorg. Chem. 2020, 59, 16016–16026. 10.1021/acs.inorgchem.0c02572. 33086004 PMC8015230

[ref16] Zacchini S. Using Metal Carbonyl Clusters To Develop a Molecular Approach towards Metal Nanoparticles. Eur. J. Inorg. Chem. 2011, 4125–4145. 10.1002/ejic.201100462.

[ref17] Muetterties E. L.; Rhodin T. N.; Band E.; Brucker C. F.; Pretzer W. R. Clusters and Surfaces. Chem. Rev. 1979, 79, 91–137. 10.1021/cr60318a001.

[ref18] Muetterties E. L.; Stein J. Mechanistic Features of Catalytic Carbon Monoxide Hydrogenation Reactions. Chem. Rev. 1979, 79, 479–490. 10.1021/cr60322a001.

[ref19] Maitlis P. M.; Zanotti V. The role of electrophilic species in the Fischer-Tropsch reaction. Chem. Commun. 2009, 1619–1634. 10.1039/b822320n. 19294244

[ref20] Liu Q.-Y.; Shang C.; Liu Z.-P. In Situ Active Site for CO Activation in Fe-Catalyzed Fischer-Tropsch Synthesis from Machine Learning. J. Am. Chem. Soc. 2021, 143, 11109–11120. 10.1021/jacs.1c04624. 34278799

[ref21] Ohata J.; Teramoto A.; Fujita H.; Takemoto S.; Matsuzaka H. Linear Hydrocarbon Chain Growth From a Molecular Diruthenium Carbide Platform. J. Am. Chem. Soc. 2021, 143, 16105–16112. 10.1021/jacs.1c06586. 34524798

[ref22] Jiao Y.; Ma H.; Wang H.; Li Y.-W.; Wen X.-D.; Jiao H. Interactive network of the dehydrogenation of alkanes, alkenes and alkynes – surface carbon hydrogenative coupling on Ru(111). Catal. Sci. Technol. 2021, 11, 191–210. 10.1039/d0cy02055a.

[ref23] Schulz H. Short history and present trends of Fischer-Tropsch synthesis. Appl. Catal., A 1999, 186, 3–12. 10.1016/s0926-860x(99)00160-x.

[ref24] West N. M.; Miller A. J. M.; Labinger J. A.; Bercaw J. E. Homogeneous Syngas Conversion. Coord. Chem. Rev. 2011, 255, 881–898. 10.1016/j.ccr.2010.08.019.

[ref25] Pattanayak S.; Berben L. A. Pre-Equilibrium Reaction Mechanism as a Strategy to Enhance Rate and Lower Overpotential in Electrocatalysis. J. Am. Chem. Soc. 2023, 145, 3419–3426. 10.1021/jacs.2c10942. 36734988 PMC9936576

[ref26] Pattanayak S.; Berben L. A. Cobalt Carbonyl Clusters Enable Independent Control of Two Proton Transfer Rates in the Mechanism for Hydrogen Evolution. ChemElectroChem 2021, 8, 2488–2494. 10.1002/celc.202100402.

[ref27] Carr C. R.; Taheri A.; Berben L. A. Fast Proton Transfer and Hydrogen Evolution Reactivity Mediated by [Co_13_C_2_(CO)_24_]^4–^. J. Am. Chem. Soc. 2020, 142, 12299–12305. 10.1021/jacs.0c04034. 32571013

[ref28] Loewen N. D.; Neelakantan T. V.; Berben L. A. Renewable Formate C-H Bond Formation with CO_2_: Using Iron Carbonyl Clusters as Electrocatalysts. Acc. Chem. Res. 2017, 50, 2362–2370. 10.1021/acs.accounts.7b00302. 28836757

[ref29] Taheri A.; Berben L. A. Tailoring Electrocatalysts for Selective CO_2_ and H^+^ Reduction: Iron Carbonyl Clusters as a Case Study. Inorg. Chem. 2016, 55, 378–385. 10.1021/acs.inorgchem.5b02293. 26689238

[ref30] Lancaster K. M.; Roemelt M.; Ettenhuber P.; Hu Y.; Ribbe M. W.; Neese F.; Bergmann U.; DeBeer S. X-ray Emission Spectroscopy Evidences a Central Carbon in the Nitrogenase Iron-Molybdenum Cofactor. Science 2011, 334, 974–977. 10.1126/science.1206445. 22096198 PMC3800678

[ref31] Spatzal T.; Aksoyoglu M.; Zhang L.; Andrade S. L. A.; Schleicher E.; Weber S.; Rees D. C.; Einsle O. Evidence for Interstitial Carbon in Nitrogenase FeMo Cofactor. Science 2011, 334, 940 10.1126/science.1214025. 22096190 PMC3268367

[ref32] Hu Y.; Ribbe M. W. Nitrogenases—A Tale of Carbon Atom(s). Angew. Chem., Int. Ed. 2016, 55, 8216–8226. 10.1002/anie.201600010. 27206025

[ref33] Buscagan T. M.; Perez K. A.; Maggiolo A. O.; Rees D. C.; Spatzal T. Structural Characterization of Two CO Molecules Bound to the Nitrogenase Active Site. Angew. Chem., Int. Ed. 2021, 60, 5704–5707. 10.1002/anie.202015751. PMC7920927 33320413

[ref34] Joseph C.; Cobb C. R.; Rose M. J. Single-Step Sulfur Insertions into Iron Carbide Carbonyl Clusters: Unlocking the Synthetic Door to FeMoco Analogues. Angew. Chem., Int. Ed. 2021, 60, 3433–3437. 10.1002/anie.202011517. 33089646

[ref35] McGale J.; Cutsail G. E.; Joseph C.; Rose M. J.; Debeer S. Spectroscopic X-ray and Mössbauer Characterization of M_6_ and M_5_ Iron(Molybdenum)-Carbonyl Carbide Clusters: High Carbide-Iron Covalency Enhances Local Iron Site Electron Density despite Cluster Oxidation. Inorg. Chem. 2019, 58, 12918–12932. 10.1021/acs.inorgchem.9b01870. 31553598 PMC6784818

[ref36] Kuppuswamy S.; Wofford J. D.; Joseph C.; Xie Z.-L.; Ali A. K.; Lynch V. M.; Lindahl P. A.; Rose M. J. Structures, Interconversions, and Spectroscopy of Iron Carbonyl Clusters with an Interstitial Carbide: Localized Metal Center Reduction by Overall Cluster Oxidation. Inorg. Chem. 2017, 56, 5998–6012. 10.1021/acs.inorgchem.7b00741. 28441025 PMC6526005

[ref37] Liu L.; Woods T. J.; Rauchfuss T. B. Reactions of [Fe_6_C(CO)_14_(S)]^2–^: Cluster Growth, Redox, Sulfiding. Eur. J. Inorg. Chem. 2020, 3460–3465. 10.1002/ejic.202000736. 33883972 PMC8054992

[ref38] Liu L.; Rauchfuss T. B.; Woods T. J. Iron Carbide-Sulfide Carbonyl Clusters. Inorg. Chem. 2019, 58, 8271–8274. 10.1021/acs.inorgchem.9b01231. 31184487 PMC6602809

[ref39] Moraru I. T.; Martínez-Prieto L. M.; Coppel Y.; Chaudret B.; Cusinato L.; del Rosal I.; Poteau R. A combined theoretical/experimental study highlighting the formation of carbides on Ru nanoparticles during CO hydrogenation. Nanoscale 2021, 13, 6902–6915. 10.1039/d0nr08735a. 33885491

[ref40] Adams R. D.; Trufan E. Ruthenium-tin cluster complexes and their applications as bimetallic nanoscale heterogeneous hydrogenation catalysts. Philos. Trans. R. Soc., A 2010, 368, 1473–1493. 10.1098/rsta.2009.0277. 20156832

[ref41] Johnson B. F. G.; Raynor S. A.; Brown D. B.; Shephard D. S.; Mashmeyer T.; Thomas J. M.; Hermans S.; Raja R.; Sankar G. New catalysts for clean technology. J. Mol. Catal. A: Chem. 2002, 182–183, 89–97. 10.1016/s1381-1169(01)00490-3.

[ref42] Thomas J. M.; Johnson B. F. G.; Raja R.; Sankar G.; Midgley P. A. High-Performance Nanocatalysts for Single-Step Hydrogenations. Acc. Chem. Res. 2003, 34, 20–30. 10.1002/chin.200316294. 12534301

[ref43] Koh A. C. W.; Leong W. K.; Chen L.; Ang T. P.; Lin J.; Johnson B. F. G.; Khimyak T. Highly efficient ruthenium and ruthenium-platinum cluster-derived nanocatalysts for hydrogen production via ethanol steam reforming. Catal. Commun. 2008, 9, 170–175. 10.1016/j.catcom.2007.05.034.

[ref44] Hungria A. B.; Raja R.; Adams R. D.; Captain B. K.; Thomas J. M.; Midgley P. A.; Golovko V.; Johnson B. F. G. Single-Step Conversion of Dimethyl Terephtalate into Cyclohexanedimethanol with Ru_5_PtSn, a Trimetallic Nanoparticle Catalyst. Angew. Chem., Int. Ed. 2006, 45, 4782–4785. 10.1002/anie.200600359. 16795100

[ref45] Uffalussy K. J.; Captain B. K.; Adams R. D.; Hungria A. B.; Monnier J. R.; Amiridis M. D. Synthesis and Characterization of Cluster-Derived PtRu_5_Sn Catalysts. ACS Catal. 2011, 1, 1710–1718. 10.1021/cs2003559.

[ref46] Nakajima T.; Ishiguro A.; Wakatsuki Y. Formation of Super Wires of Clusters by Self-Assembly of Transition Metal Cluster Anions with Metal Cations. Angew. Chem., Int. Ed. 2001, 40, 1066–1068. 10.1002/1521-3773(20010316)40:6<1066::aid-anie10660>3.0.co;2-1. 11268075

[ref47] Nakajima T.; Konomoto H.; Ogawa H.; Wakatsuki Y. Synthesis of three-component high nuclearity cluster complexes with ruthenium carbido carbonyl clusters as a building block. J. Organomet. Chem. 2007, 692, 5071–5080. 10.1016/j.jorganchem.2007.07.054.

[ref48] Khimyak T.; Johnson B. F. G.; Hermans S.; Bond A. D. The synthesis and characterisation of the cluster dianion [PtRu_5_C(CO)_15_]^2–^ and its reactions with Au and Pt cationic fragments produced in situ. Dalton Trans. 2003, 2651–2657. 10.1039/b303178k.

[ref49] Koshevoy I. O.; Haukka M.; Pakkanen T. A.; Tunik S. P. Synthesis of nonanuclear heterometallic carbide clusters. Unexpected formation of the [Ru_6_(CO)_16_]^2–^[Pt_2_(CO)_2_(dppm)_2_]^2+^ ion pair on the way to [Ru_6_C(CO)_16_Pt_3_(dppm)_2_]. Dalton Trans. 2006, 5641–5647. 10.1039/b609253e. 17225900

[ref50] Johnson B. F. G.; Lewis J.; Sankey S. W.; Wong K.; McPartlin M.; Nelson W. J. H. An improved synthesis of the hexaruthenium carbido cluster Ru_6_C(CO)_17_; X-ray structure of the salt [Ph_4_As]_2_[Ru_6_C(CO)_16_]. J. Organomet. Chem. 1980, 191, C3–C7. 10.1016/s0022-328x(00)81077-3.

[ref51] Chihara T.; Yamazaki H. Synthesis and Structural Characterization of the Hydrido Carbido Ruthenium Cluster [PPh_4_] [Ru_6_C(CO)_16_H]. J. Cluster Sci. 1992, 3, 489–497. 10.1007/bf00702754.

[ref52] Hayward C.-M. T.; Shapley J. R. Systematic and Efficient Synthesis of Ru_6_(CO)_18_^2–^, Ru_6_C(CO)_16_^2–^, Os_6_(CO)_18_^2–^, Os_10_C(CO)_24_^2–^. Isolation and Characterization of Os_6_C(CO)_17_. Inorg. Chem. 1982, 21, 3816–3820. 10.1021/ic00140a043.

[ref53] Chihara T.; Yamazaki H. Hexaruthenium carbido carbonyl methyl cluster [PPN] [Ru_6_C(CO)_16_(CH_3_)] as catalyst precursor for hydrogenation of olefins. Synthesis and structures of unsaturated and saturated hexaruthenium hydrido clusters [PPN] [Ru_6_C(CO)_15_H] and [PPN] [Ru_6_C(CO)_16_H]. J. Organomet. Chem. 1994, 473, 273–284. 10.1016/0022-328x(94)80128-2.

[ref54] Li C.; Xu J.; Zhao J.; Tian D.; King R. B. The maximum number of carbonyl groups around an Ru_6_C polyhedral cluster: hexanuclear ruthenium carbonyl carbides. Dalton Trans. 2010, 39, 10697–10701. 10.1039/c0dt00670j. 20941429

[ref55] Mingos D. M. P. Polyhedral Skeletal Electron Pair Approach. Acc. Chem. Res. 1984, 17, 311–319. 10.1021/ar00105a003.

[ref56] Bortoluzzi M.; Ciabatti I.; Cesari C.; Femoni C.; Iapalucci M. C.; Zacchini S. Synthesis of the Highly Reduced [Fe_6_C(CO)_15_]^4–^ Carbonyl Carbide Cluster and its Reactions with H^+^ and [Au(PPh_3_)]^+^. Eur. J. Inorg. Chem. 2017, 3134–3143. 10.1002/ejic.201700564.

[ref57] Kabir S. E.; Day M.; Irving M.; McPhillips T.; Minassian H.; Rosenberg E.; Hardcastle K. I. Reactions of Bis(acetonitrile)triosmium Decacarbonyl with Secondary Mixed Amines NHRR’ (R = CH_2_CH_3_, R’ = CH_3_ or n-CH_2_CH_2_CH_3_). Organomet 1991, 10, 3997–4004. 10.1021/om00058a014.

[ref58] Drake S. R.; Johnson B. F. G.; Lewis J. Redox activation of the hexanuclear ruthenium cluster [Ru_6_C(CO)_16_]^2–^. J. Chem. Soc., Dalton Trans. 1989, 243–246. 10.1039/dt9890000243.

[ref59] Krejčik M.; Daněk M.; Hartl F. Simple construction of an infrared optically transparent thin-layer electrochemical cell: Applications to the redox reactions of ferrocene, Mn_2_(CO)_10_ and Mn(CO)_3_(3,5-di-t-butyl-catecholate). J. Electroanal. Chem. 1991, 317, 179–187. 10.1016/0022-0728(91)85012-e.

[ref60] Cesari C.; Bortoluzzi M.; Femoni C.; Carmela Iapalucci M.; Zacchini S. Synthesis, molecular structure and fluxional behavior of the elusive [HRu_4_(CO)_12_]^3–^ carbonyl anion. Dalton Trans. 2022, 51, 2250–2261. 10.1039/d1dt03622j. 35060580

[ref61] Keller E. SCHAKAL99; University of Freiburg: Freiburg, Germany, 1999.

[ref62] Sheldrick G. M. SADABS-2008/1-Bruker AXS Area Detector Scaling and Absorption Correction; Bruker AXS: Madison, WI, 2008.

[ref63] Sheldrick G. M. Crystal structure refinement withSHELXL. Acta Crystallogr., Sect. C: Struct. Chem. 2015, 71, 3–8. 10.1107/s2053229614024218. 25567568 PMC4294323

[ref64] Spek A. L. Single-crystal structure validation with the program PLATON. J. Appl. Crystallogr. 2003, 36, 7–13. 10.1107/s0021889802022112.

[ref65] Spek A. L. Structure validation in chemical crystallography. Acta Crystallogr., Sect. D: Biol. Crystallogr. 2009, 65, 148–155. 10.1107/s090744490804362x. 19171970 PMC2631630

[ref66] Grimme S.; Brandenburg J. G.; Bannwarth C.; Hansen A. A. Consistent structures and interactions by density functional theory with small atomic orbital basis sets. J. Chem. Phys. 2015, 143, 054107 10.1063/1.4927476. 26254642

[ref67] Otlyotov A. A.; Moshchenkov A. D.; Cavallo L.; Minenkov Y. 16OSTM10: a new open-shell transition metal conformational energy database to challenge contemporary semiempirical and force field methods. Phys. Chem. Chem. Phys. 2022, 24, 17314–17322. 10.1039/d2cp01659a. 35815793

[ref68] Weigend F.; Ahlrichs R. Balanced basis sets of split valence, triple zeta valence and quadruple zeta valence quality for H to Rn: Design and assessment of accuracy. Phys. Chem. Chem. Phys. 2005, 7, 3297–3305. 10.1039/b508541a. 16240044

[ref69] Weigend F. Accurate Coulomb-fitting basis sets for H to Rn. Phys. Chem. Chem. Phys. 2006, 8, 1057–1065. 10.1039/b515623h. 16633586

[ref70] Kruse H.; Grimme S. A geometrical correction for the inter- and intra-molecular basis set superposition error in Hartree-Fock and density functional theory calculations for large systems. J. Chem. Phys. 2012, 136, 154101 10.1063/1.3700154. 22519309

[ref71] Grimme S.; Ehrlich S.; Goerigk L. Effect of the damping function in dispersion corrected density functional theory. J. Comput. Chem. 2011, 32, 1456–1465. 10.1002/jcc.21759. 21370243

[ref72] Grimme S.; Antony J.; Ehrlich S.; Krieg H. A consistent and accurate ab initio parametrization of density functional dispersion correction (DFT-D) for the 94 elements H-Pu. J. Chem. Phys. 2010, 132, 154104 10.1063/1.3382344. 20423165

[ref73] Cossi M.; Rega N.; Scalmani G.; Barone V. Energies, structures, and electronic properties of molecules in solution with the C-PCM solvation model. J. Comput. Chem. 2003, 24, 669–681. 10.1002/jcc.10189. 12666158

[ref74] Barone V.; Cossi M. Quantum Calculation of Molecular Energies and Energy Gradients in Solution by a Conductor Solvent Model. J. Phys. Chem. A 1998, 102, 1995–2001. 10.1021/jp9716997.

[ref75] Cramer C. J. Essentials of Computational Chemistry, 2nd ed.; Wiley: Chichester, 2004.

[ref76] Neese F. Software update: The ORCA program system - Version 5.0. Wiley Interdiscip. Rev.: Comput. Mol. Sci. 2022, 12, e1616 10.1002/wcms.1606.

[ref77] Lu T.; Chen F. Multiwfn: A Multifunctional Wavefunction Analyzer. J. Comput. Chem. 2012, 33, 580–592. 10.1002/jcc.22885. 22162017

