# Supplementary material for: Highly Reduced Ruthenium Carbide Carbonyl Clusters: Synthesis, Molecular Structure, Reactivity, Electrochemistry, and Computational Investigation of [Ru6C(CO)15]4–
Source: Inorg Chem. 2023 Aug 30;62(36):14590–603. doi: 10.1021/acs.inorgchem.3c01711 (PMC10498495; doi:10.1021/acs.inorgchem.3c01711)
Supplement: Supplementary file 1 — ic3c01711_si_001.pdf [file ic3c01711_si_001.pdf]

Supporting Information for

Highly Reduced Ruthenium Carbide Carbonyl Clusters: Synthesis, Molecular Structure, Reactivity, Electrochemistry and Computational Investigation of $[Ru_6C(CO)_{15}]^{4-}$

Cristiana Cesari,[a]* Marco Bortoluzzi,[b] Tiziana Funaioli,[c] Cristina Femoni,[a] Maria Carmela Iapalucci,[a] and Stefano Zacchini[a]

[a] Dipartimento di Chimica Industriale "Toso Montanari", Università di Bologna, Viale Risorgimento 4 - 40136 Bologna. Italy. E-mail: cristiana.cesari2@unibo.it

[b] Dipartimento di Scienze Molecolari e Nanosistemi, Ca' Foscari University of Venice, Via Torino 155 – 30175 Mestre (Ve), Italy.

[c] Dipartimento di Chimica e Chimica Industriale, Università di Pisa, Via G. Moruzzi 13 - 56124, Pisa, Italy.

	Page/s
IR spectra	S2-S6
NMR spectra	S7-S11
ESI-MS spectrum of $[Ru_6C(CO)_{14}(COCH_3)]^{3-}$ (**6**)	S12
Molecular structure of $[RuCl_3(CO)_2(CH_3CN)_2]^{-}$	S12
Supplementary electrochemical and spectroelectrochemical figures	S13-S15
Supplementary computational tables and figures	S16-S19
X-Ray crystallographic study	S20-S22

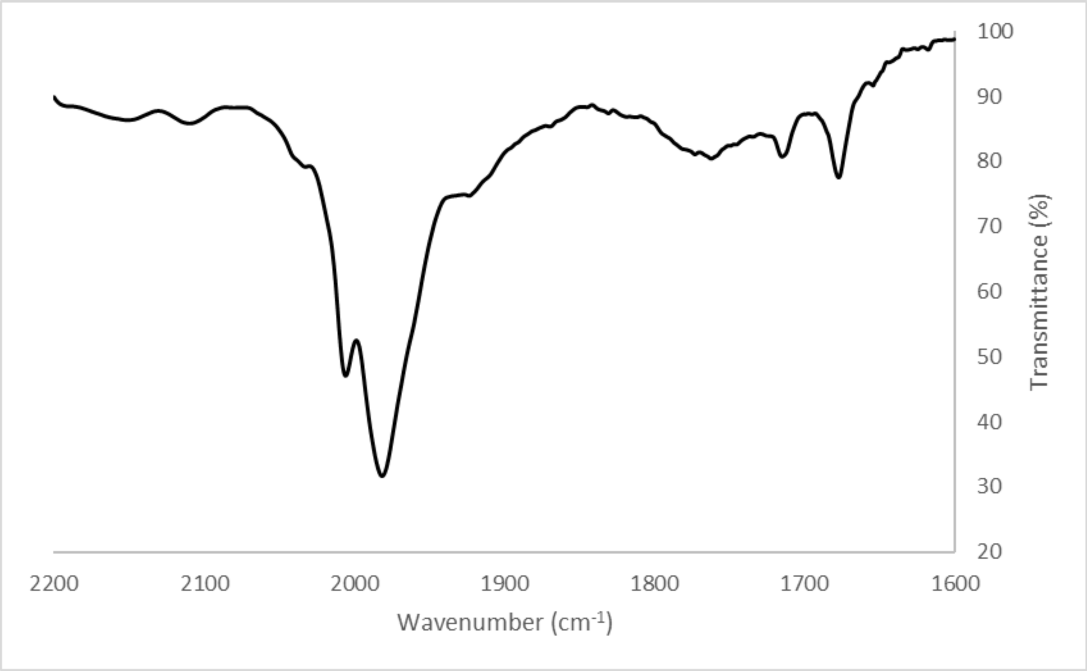

Figure S1. IR spectrum in the ν_{CO} region of $[NEt_4]_2[Ru_6C(CO)_{16}]$ (**1**) in CH_3CN.

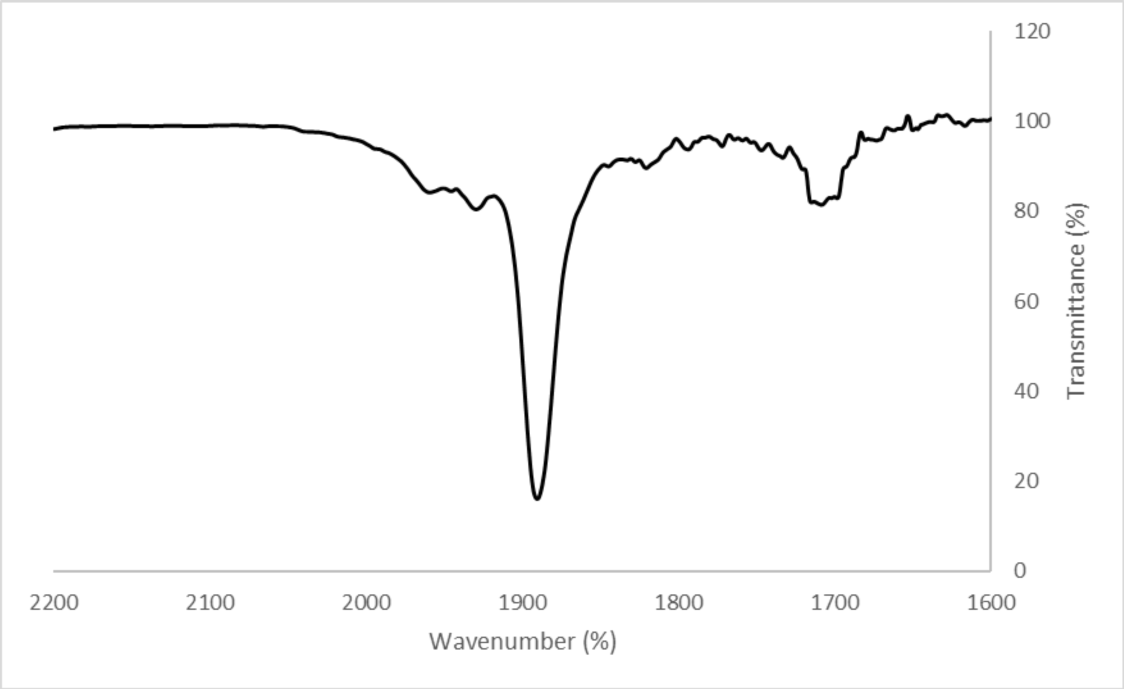

Figure S2. IR spectrum in the ν_{CO} region of $[NEt_4]_4[Ru_6C(CO)_{15}]$ (**2**) in CH_3CN.

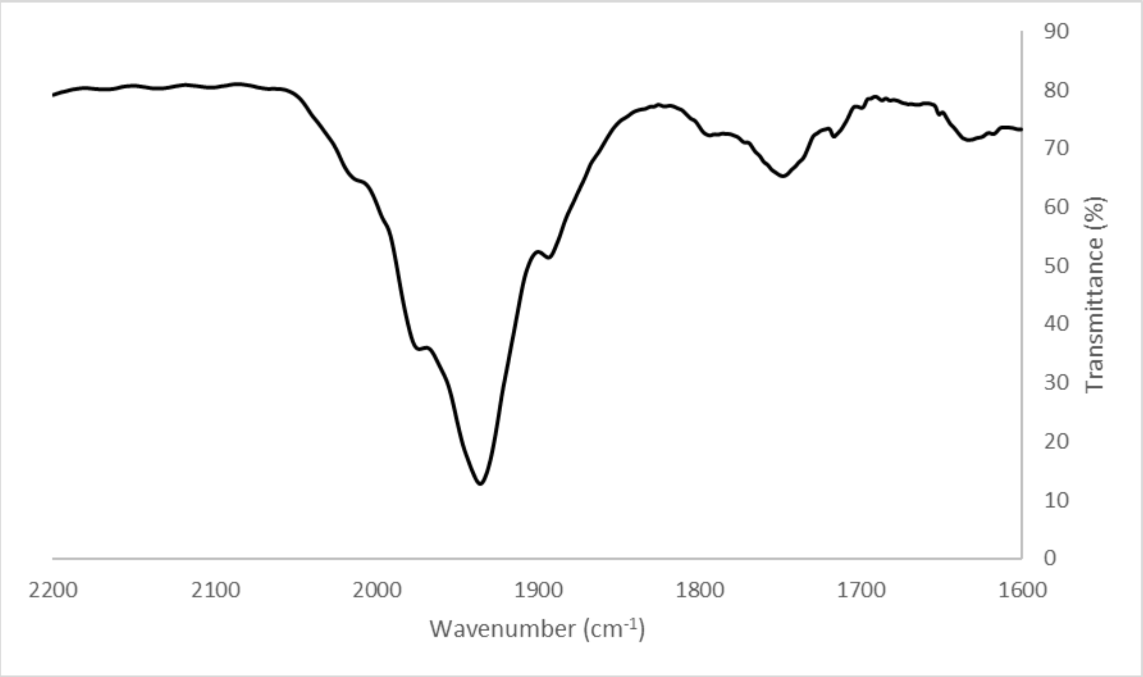

Figure S3. IR spectrum in the ν_{CO} region of $[NEt_4]_3[HRu_6C(CO)_{15}]$ (**3**) in CH_3CN.

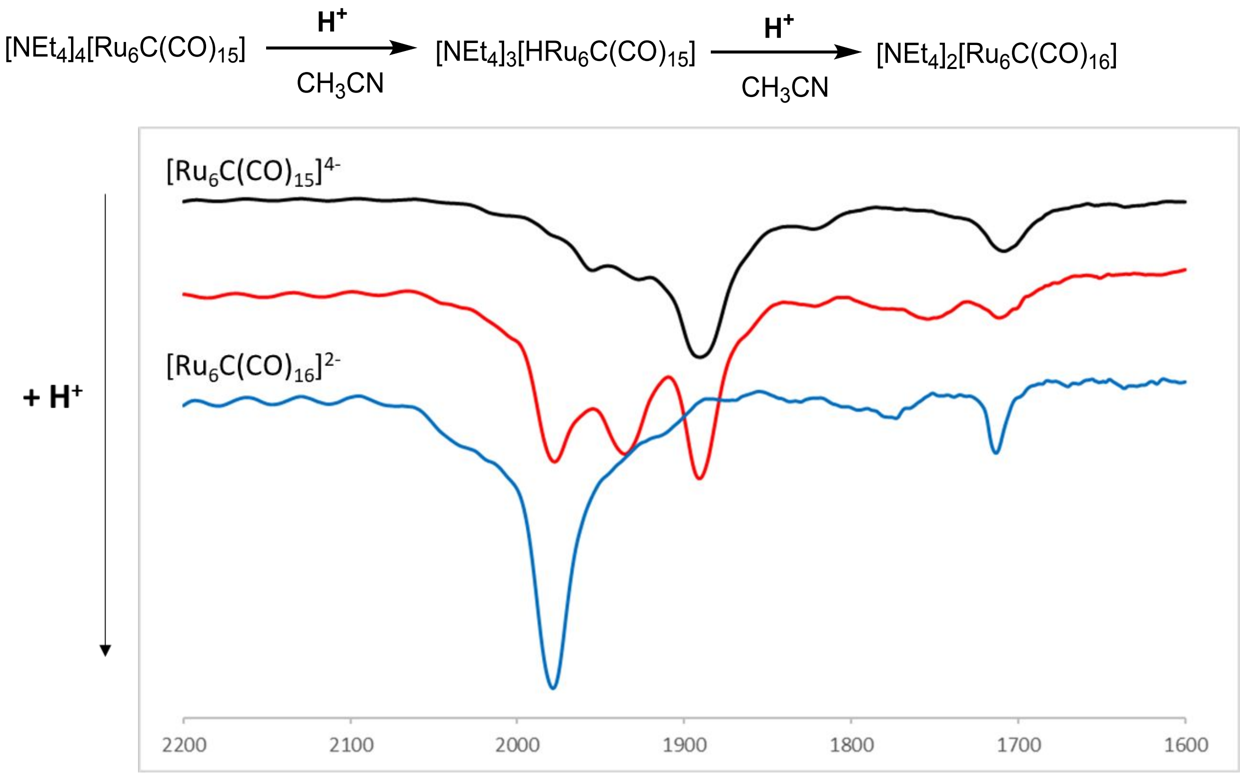

Figure S4. Reaction of $[Ru_6C(CO)_{15}]^{4-}$ (**2**) with $HBF_4{\cdot}Et_2O$ in CH_3CN followed by IR spectroscopy.

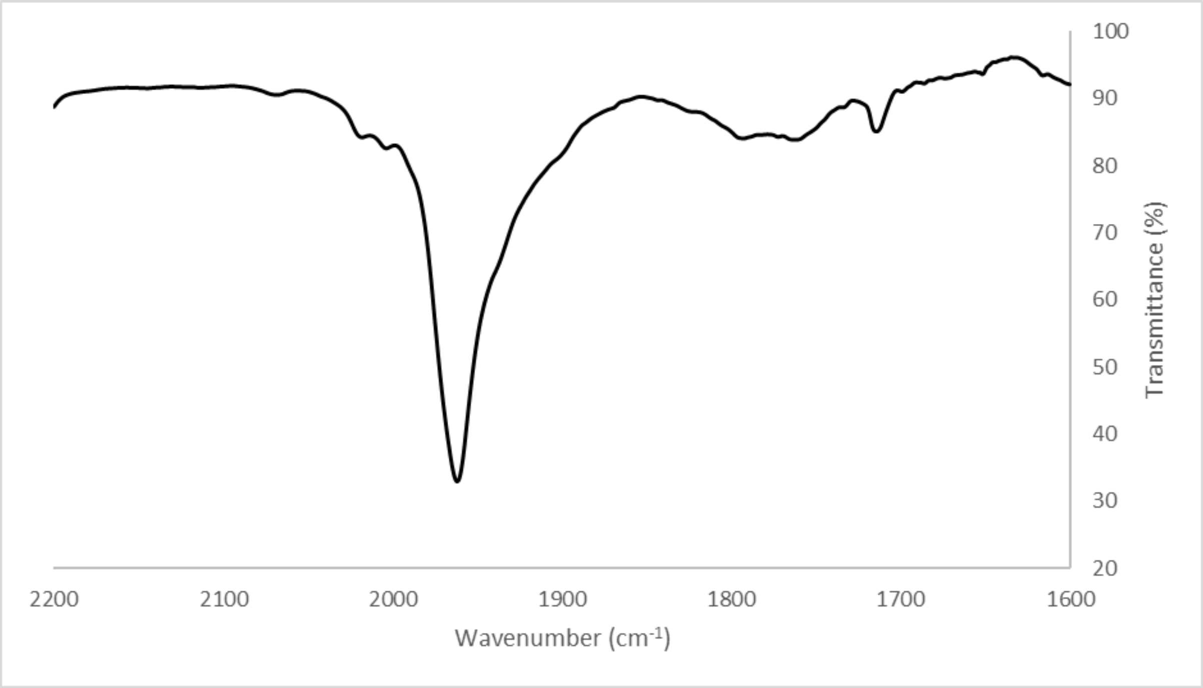

Figure S5. IR spectrum in the ν_{CO} region of $[NEt_4]_2[Ru_6C(CO)_{15}(CH_3CN)]$ (**5**) in CH_3CN.

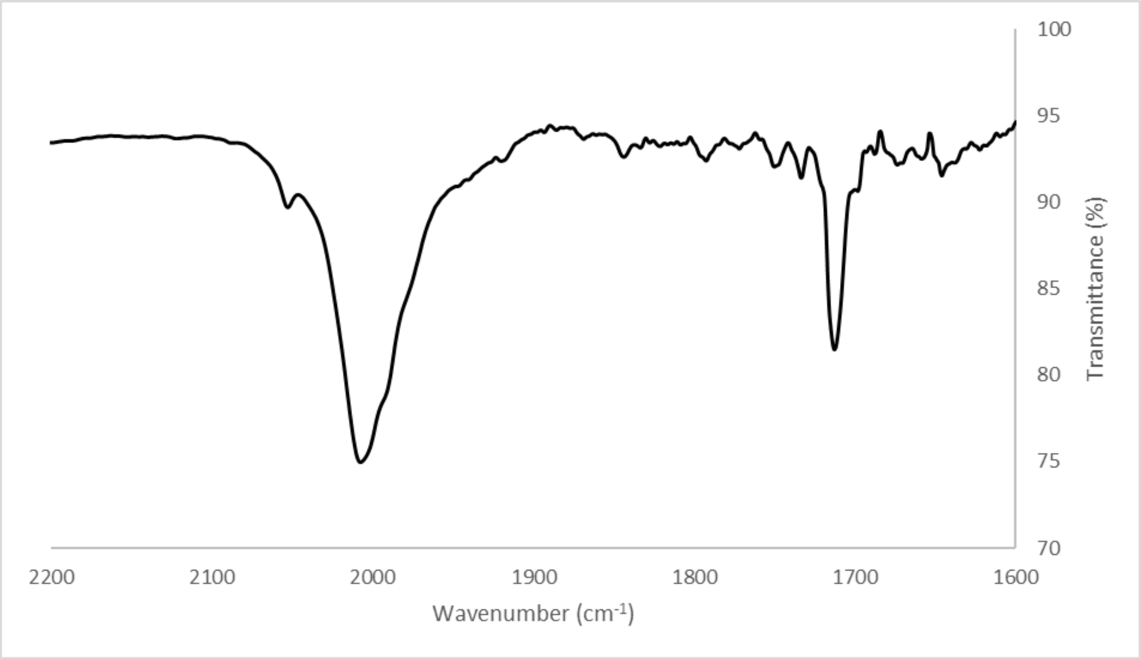

Figure S6. IR spectrum in the ν_{CO} region of $[NEt_4][HRu_6C(CO)_{16}]$ (**4**) in CH_2Cl_2.

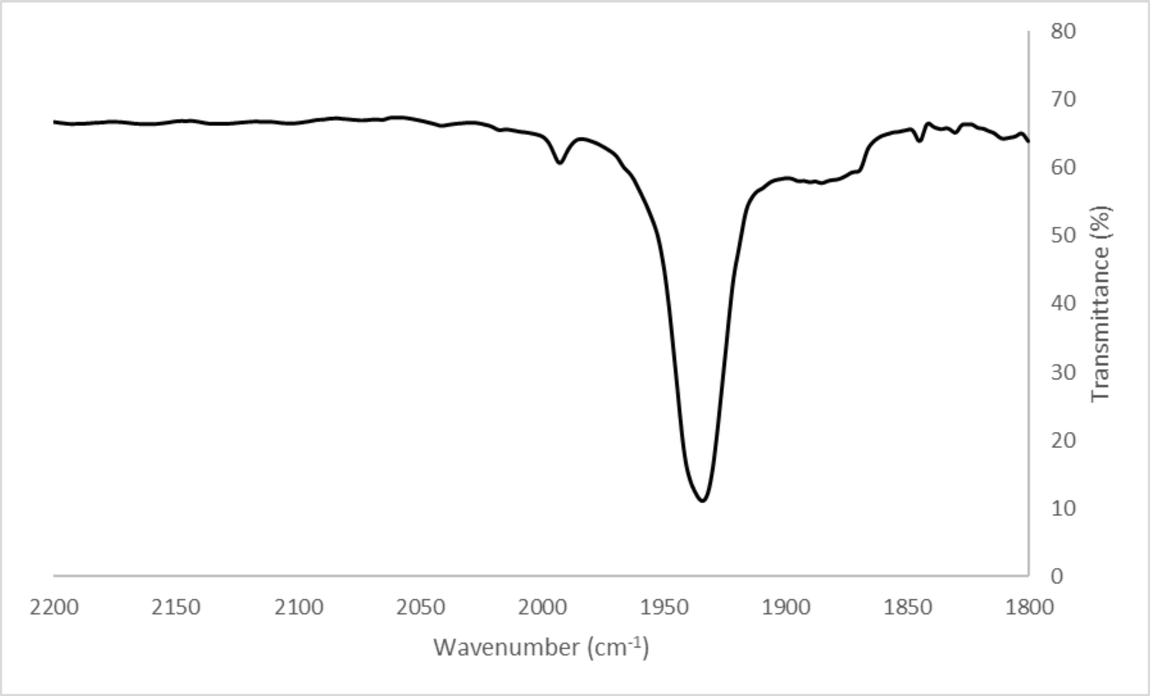

Figure S7. IR spectrum in the ν_{CO} region of the purported species $[NEt_4]_3[Ru_6C(CO)_{14}(COCH_3)]$ (**6**) in DMF.

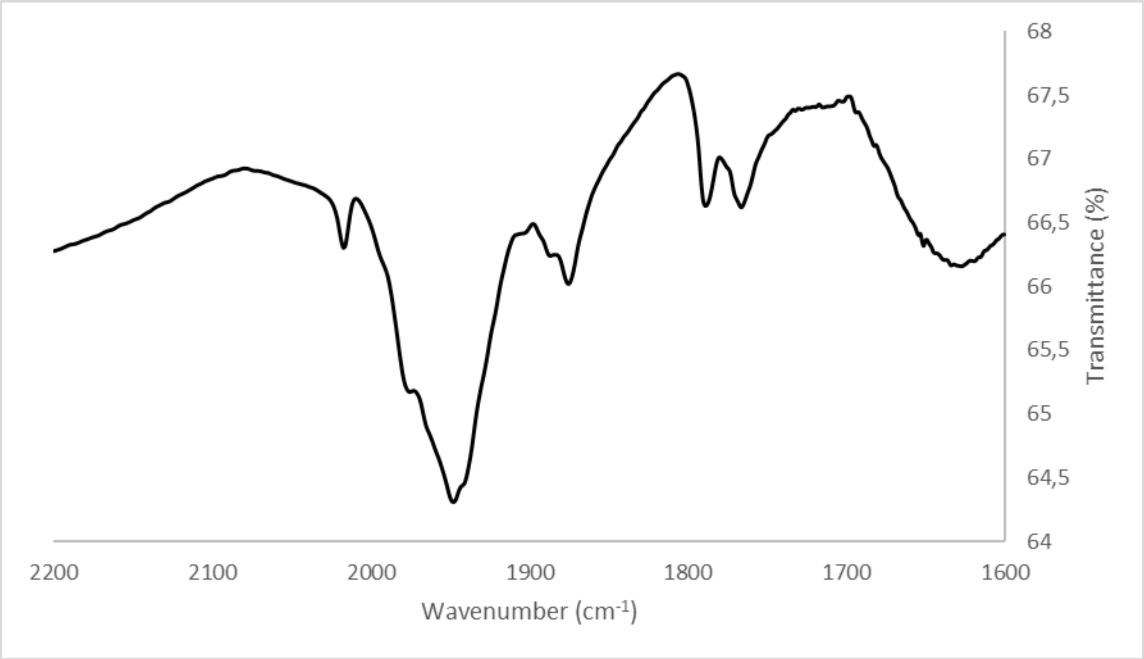

Figure S8. IR spectrum in the ν_{CO} region of the purported species $[NEt_4]_3[Ru_6C(CO)_{14}(COCH_3)]$ (**6**) in nujol.

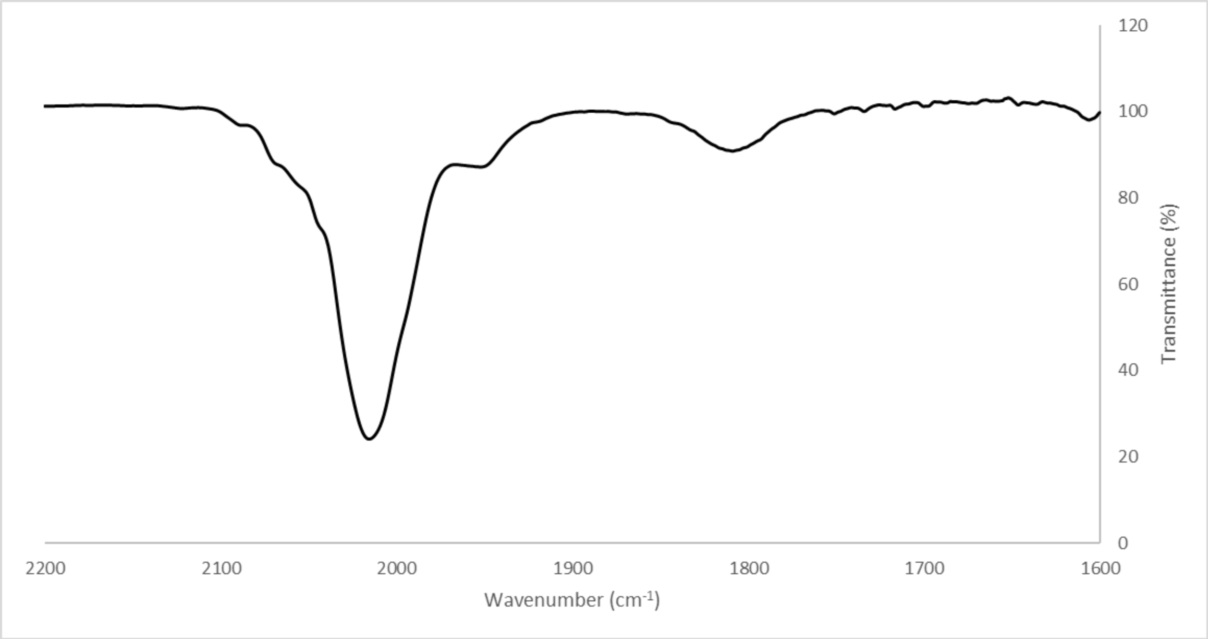

Figure S9. IR spectrum in the ν_{CO} region of $[NEt_4][H_3Ru_6C(CO)_{15}]$ (**7**) in CH_2Cl_2.

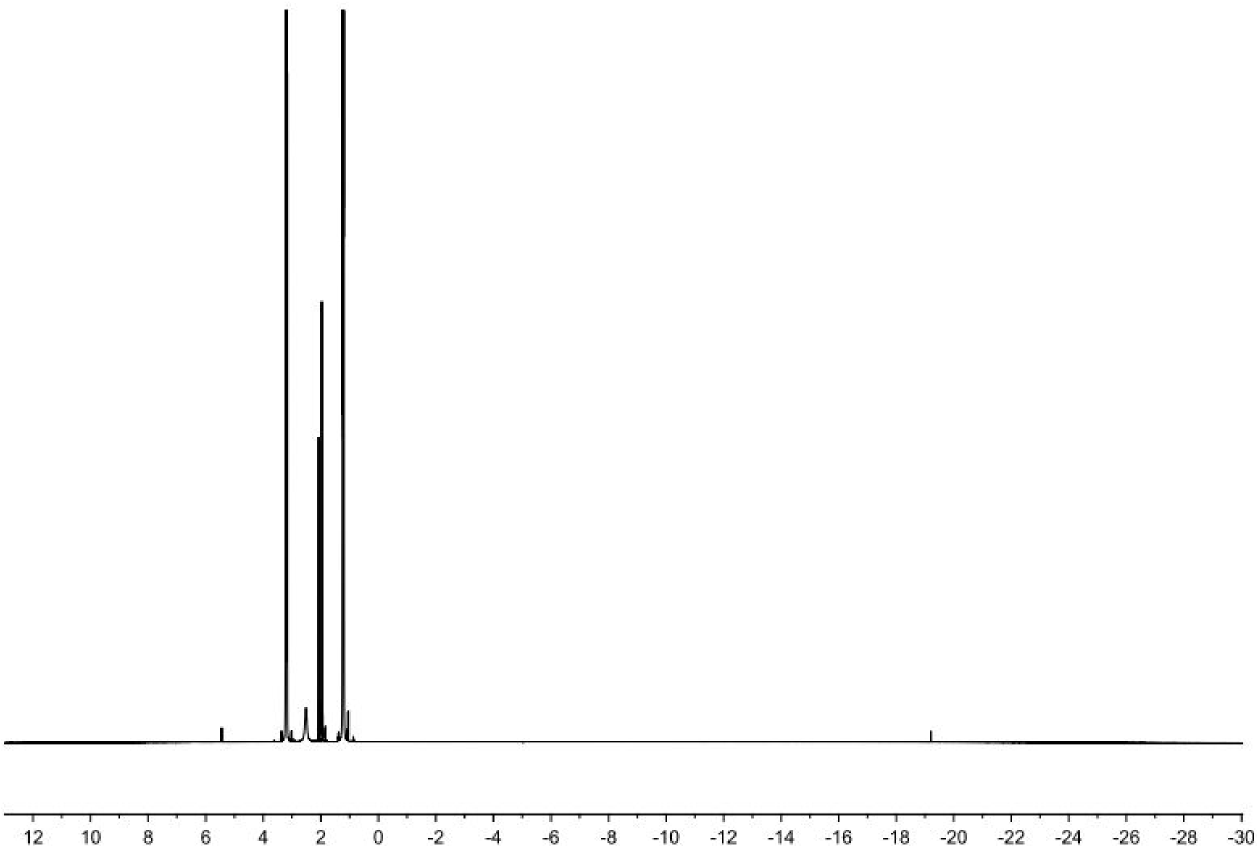

Figure S10. ^{1}H NMR spectrum of $[NEt_4]_3[HRu_6(CO)_{15}]$ (**3**) in CD_3CN at 298 K.

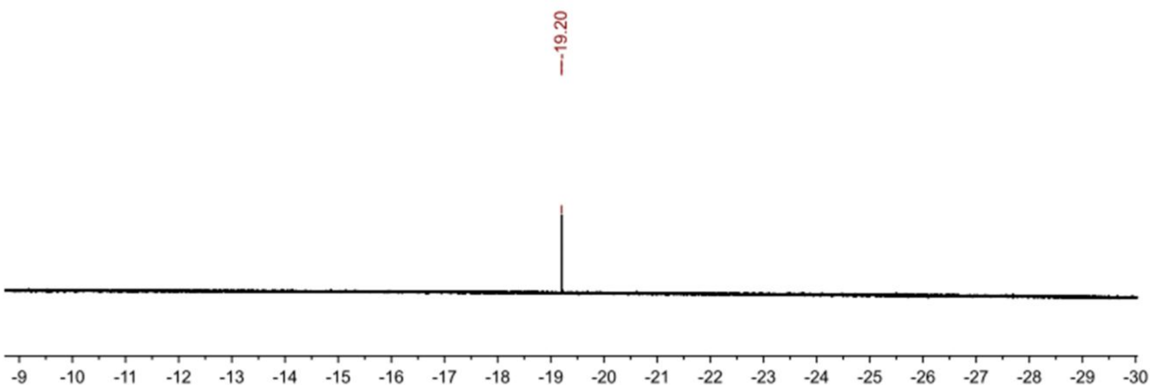

Figure S11. Hydride region of the ^{1}H NMR spectrum of $[NEt_4]_3[HRu_6(CO)_{15}]$ (**3**) in CD_3CNat 298 K.

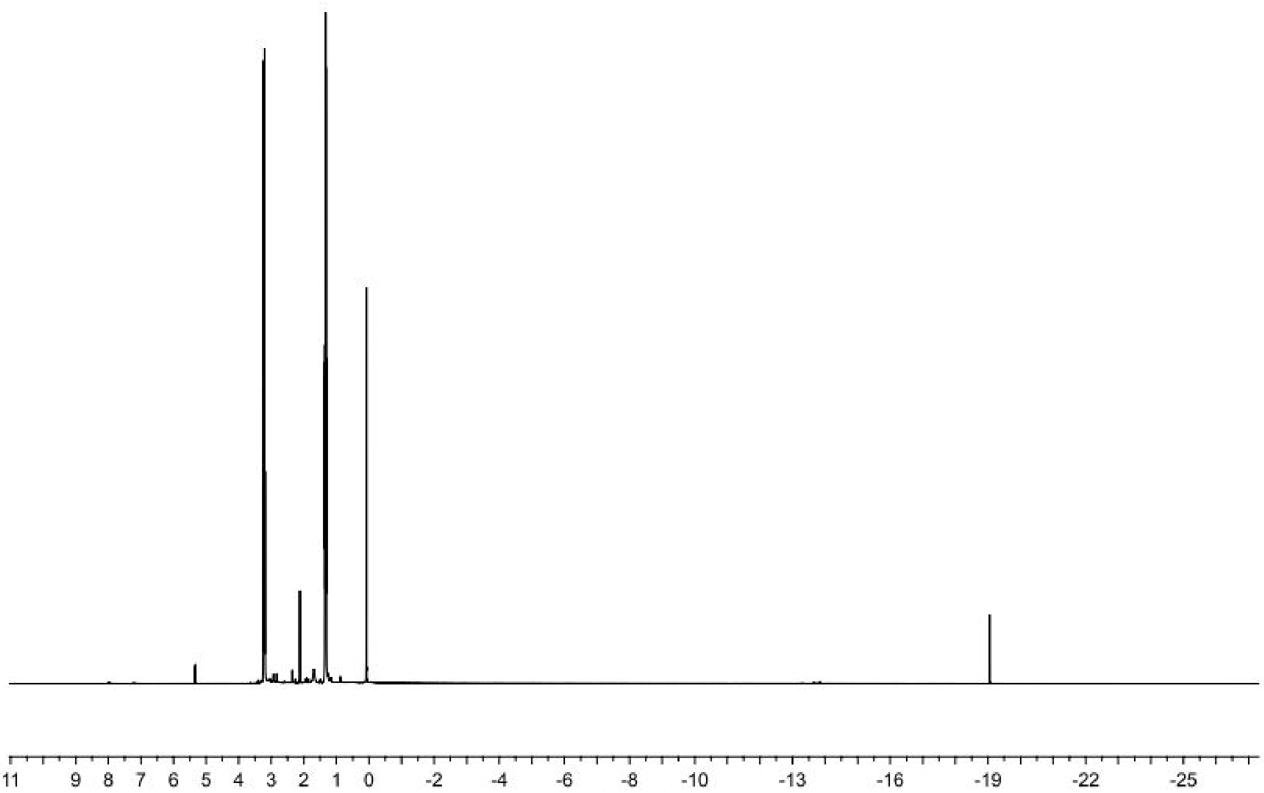

Figure S12. ^{1}H NMR spectrum of $[NEt_4][HRu_6(CO)_{16}]$ (**4**) in CD_2Cl_2 at 298 K.

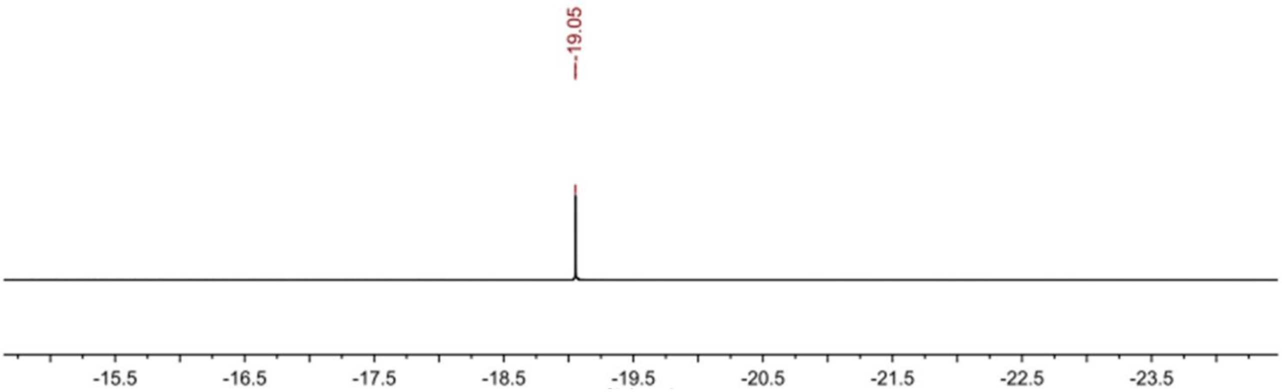

Figure S13. Hydride region of the ^{1}H NMR spectrum of $[NEt_4][HRu_6(CO)_{16}]$ (**4**) in CD_2Cl_2 at 298 K.

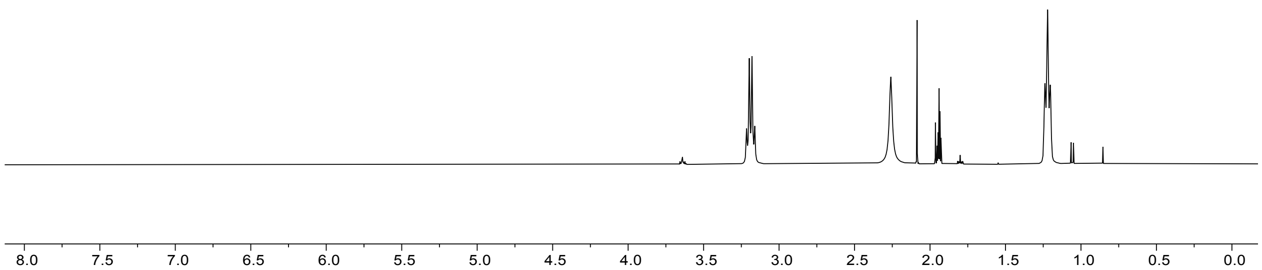

Figure S14. ^{1}H NMR spectrum of $[NEt_4]_2[Ru_6C(CO)_{15}(CH_3CN)]$ (**5**) in CD_3CN at 298 K.

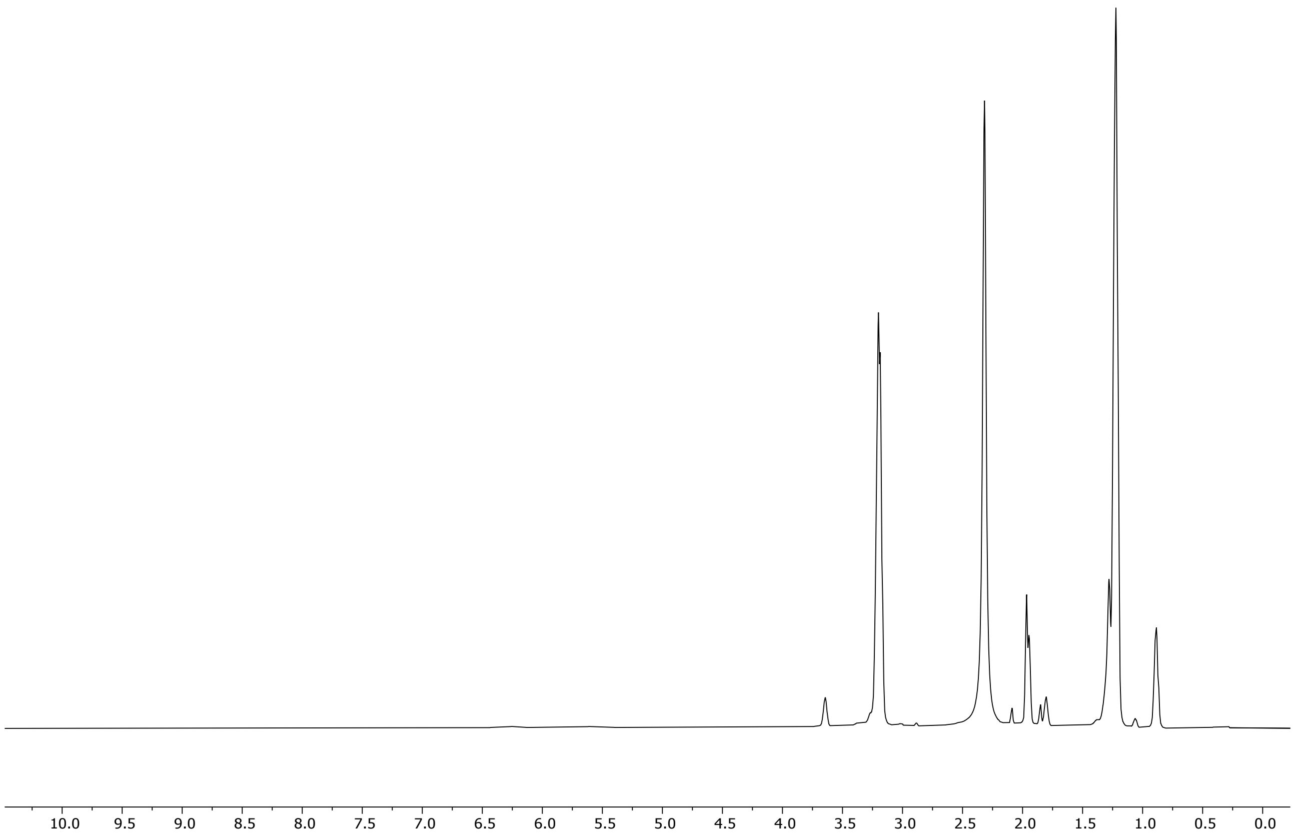

Figure S15. ^{1}H NMR spectrum of $[Ru_6C(CO)_{14}(COCH_3)]^{3-}$ (**6**) in CD_3CN at 298 K.

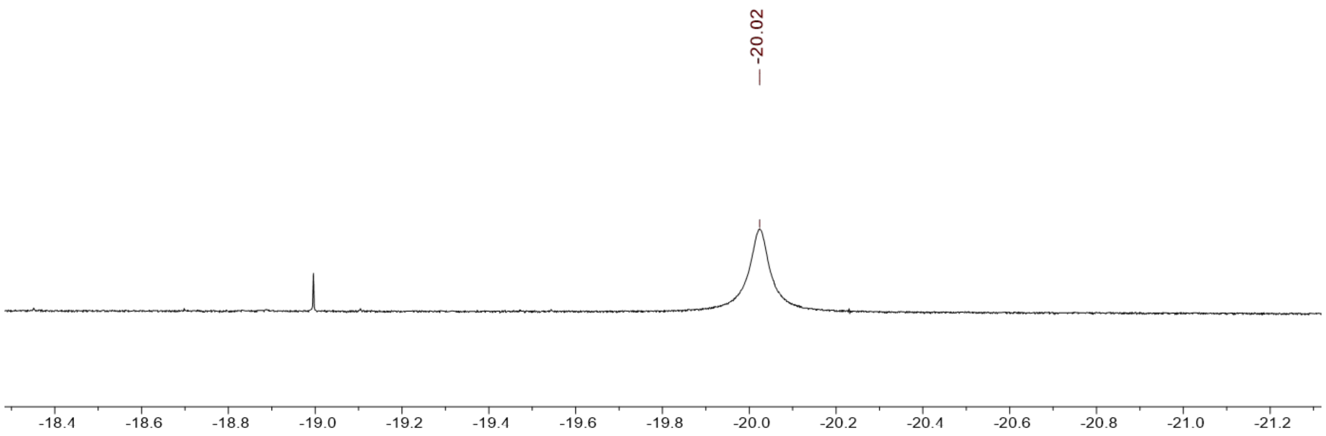

Figure S16. Hydride region of the ^{1}H NMR spectrum of $[NEt_4][H_3Ru_6(CO)_{15}]$ (**7**) in CD_2Cl_2 at 298 K.

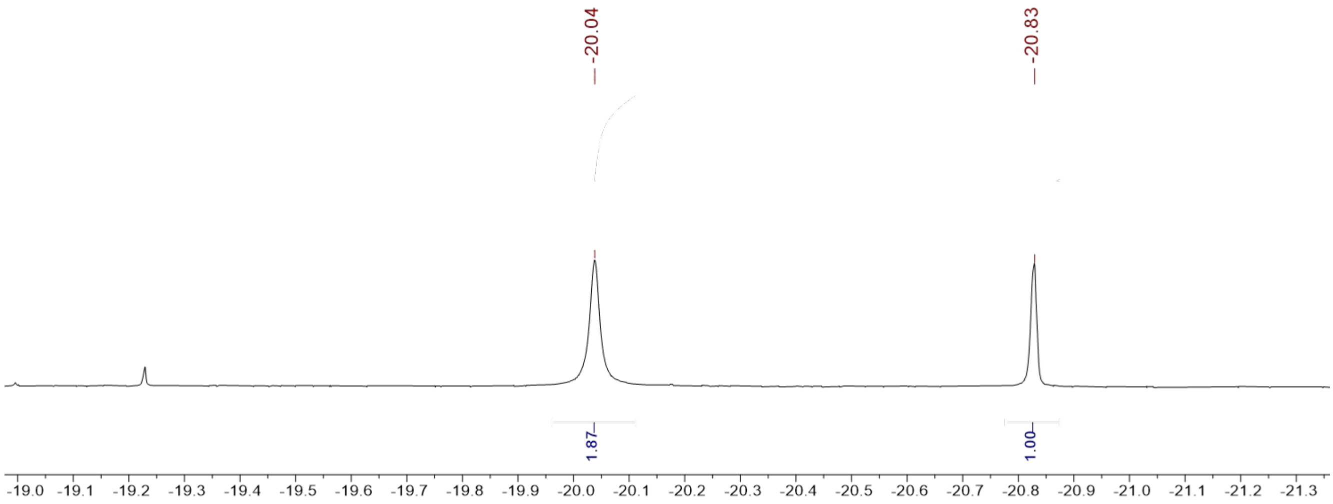

Figure S17. Hydride region of the ^{1}H NMR spectrum of $[NEt_4][H_3Ru_6(CO)_{15}]$ (**7**) in CD_2Cl_2 at 223K.

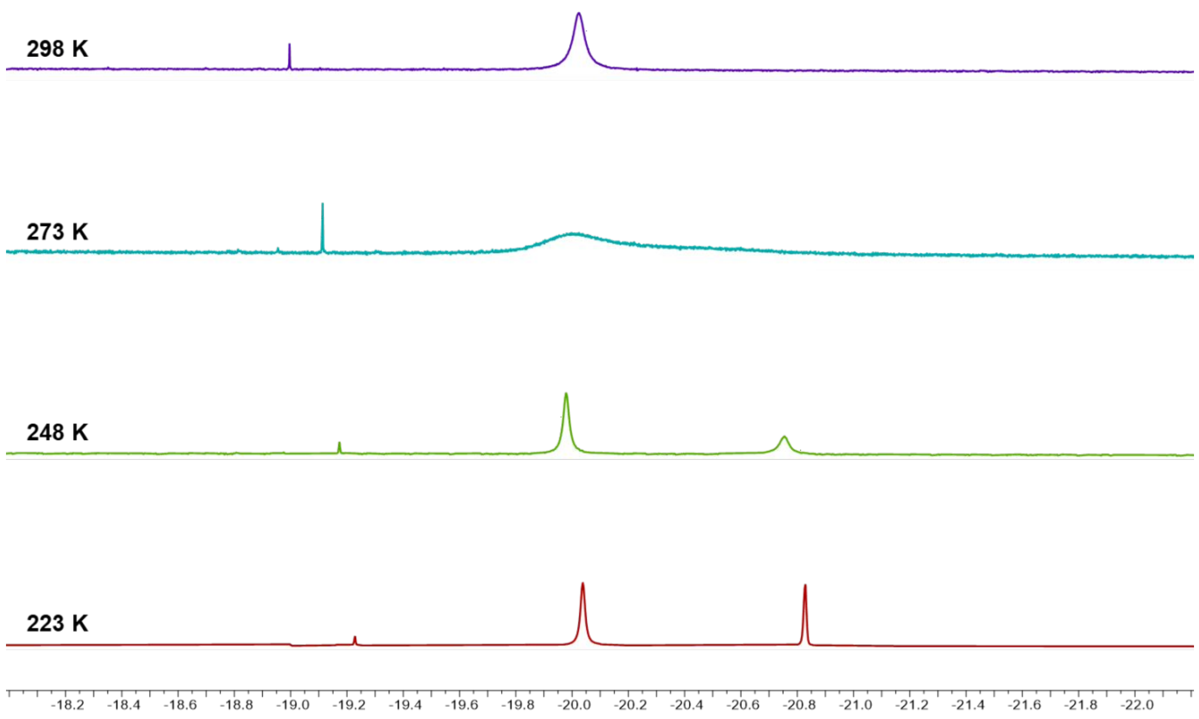

Figure S18. Hydride region of the VT ^{1}H NMR spectra of $[NEt_4][H_3Ru_6(CO)_{15}]$ (**7**) in CD_2Cl_2.

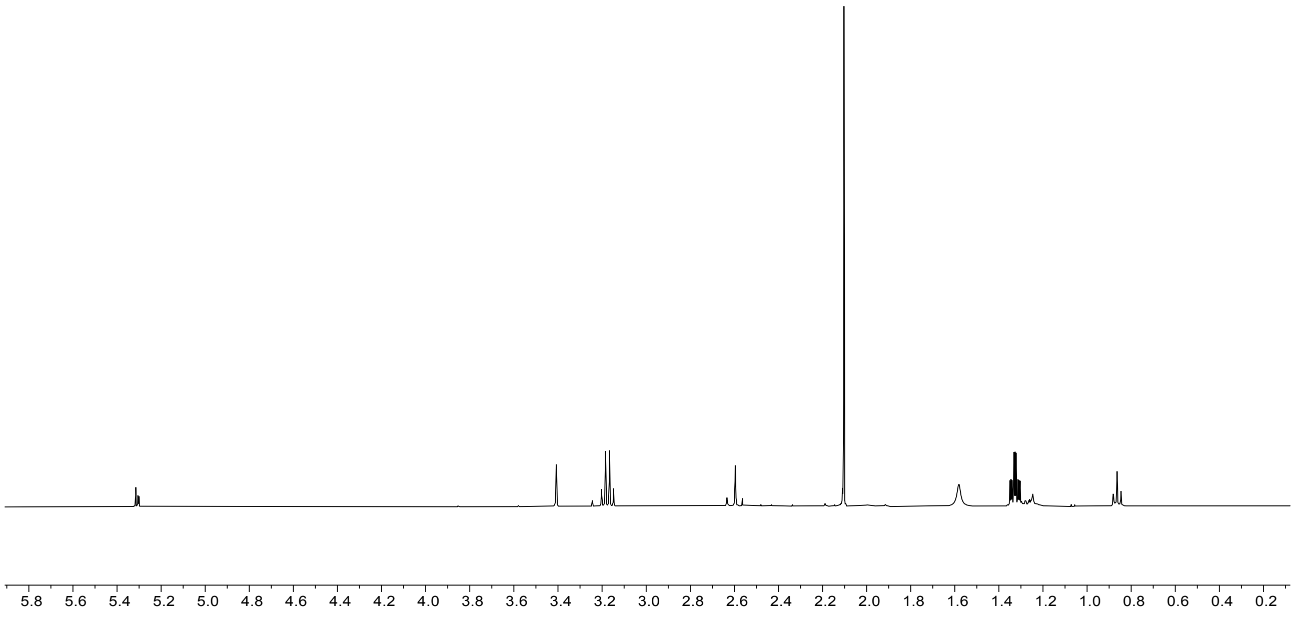

Figure S19. ^{1}H NMR spectrum of $[NEt_4][Ru_6C(CO)_{15}(CH_3CNCH_3)]$ (**8**) in CD_3CN at 298 K.

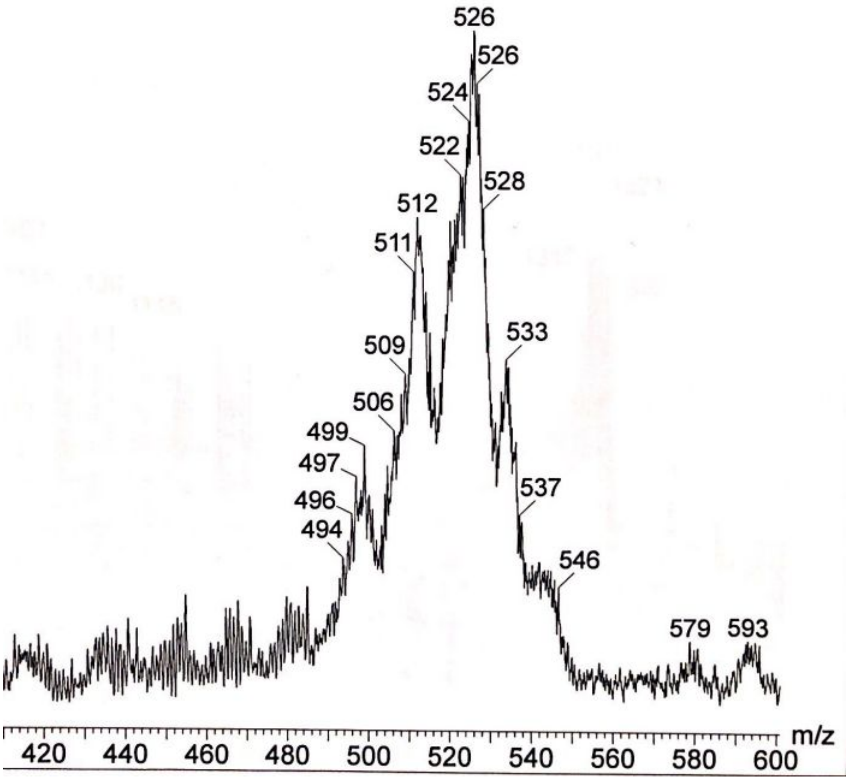

Figure S20. ESI-MS spectrum in CH_3CN (ES–) of the purported species $[Ru_6C(CO)_{14}(COCH_3)]^{3-}$ (**6**).

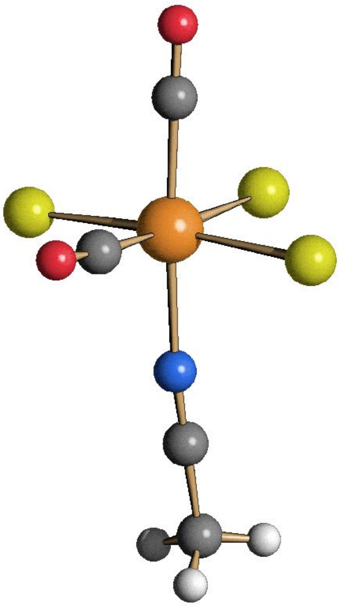

Figure S21. Molecular structure of $[RuCl_3(CO)_2(CH_3CN)_2]^-$ (orange Ru; yellow Cl; red O; blue N; grey C; white H).

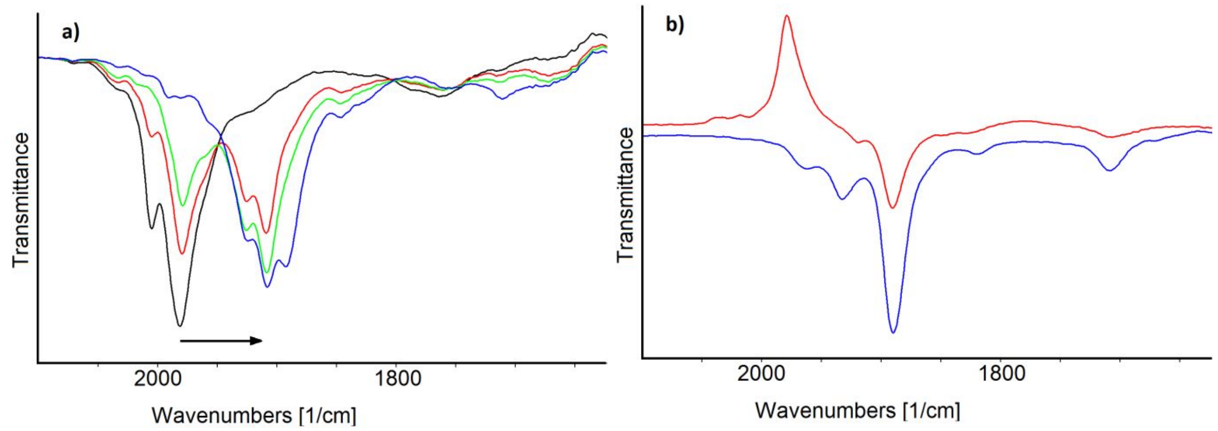

Figure S22. IR spectra of a CH_3CN solution of $[Ru_6C(CO)_{16}]^{2-}$ (**1**) recorded in an OTTLE cell: a) during the progressive decrease of the potential from –1.2 to –1.8 V (*vs* Ag pseudo-reference electrode, scan rate 2 mV sec^{-1}); b) (red line) difference spectrum between the blue and green spectra of part a) and (blue line) the spectrum of an authentic sample of $[Ru_6C(CO)_{15}]^{4-}$ (**2**). $[N^nBu_4][PF_6]$ (0.1 mol dm^{-3}) as the supporting electrolyte. The absorptions of the solvent and supporting electrolyte have been subtracted.

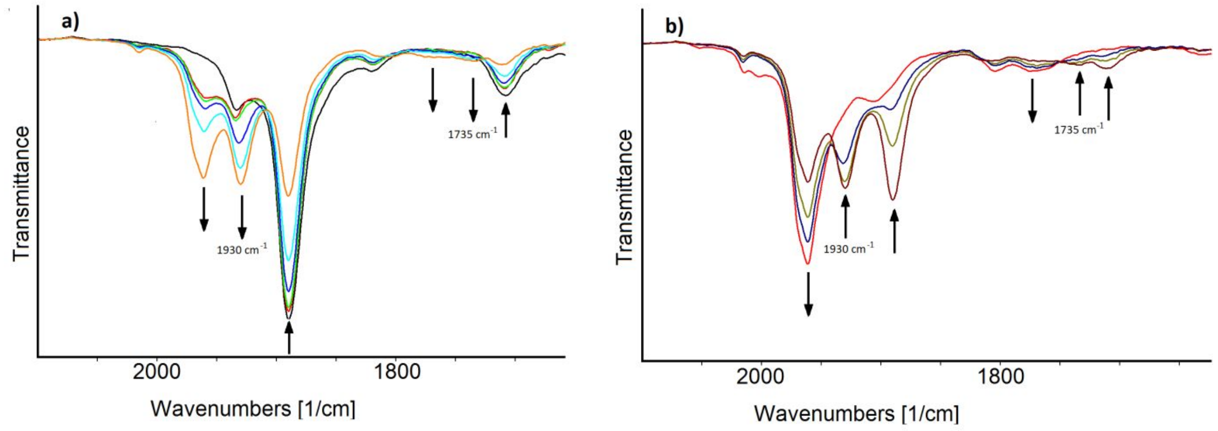

Figure S23. IR spectra of a CH_3CN solution of $[Ru_6C(CO)_{15}]^{4-}$ (**2**) recorded in an OTTLE cell during the progressive increase of the potential a) from–0.2 to +0.04 V and b) from +0.04 to +0.2 V, *vs* Ag pseudo reference electrode (scan rate 1 mV sec^{-1}). $[N^nBu_4][PF_6]$ (0.1 mol dm^{-3}) as the supporting electrolyte. The absorptions of the solvent and supporting electrolyte have been subtracted.

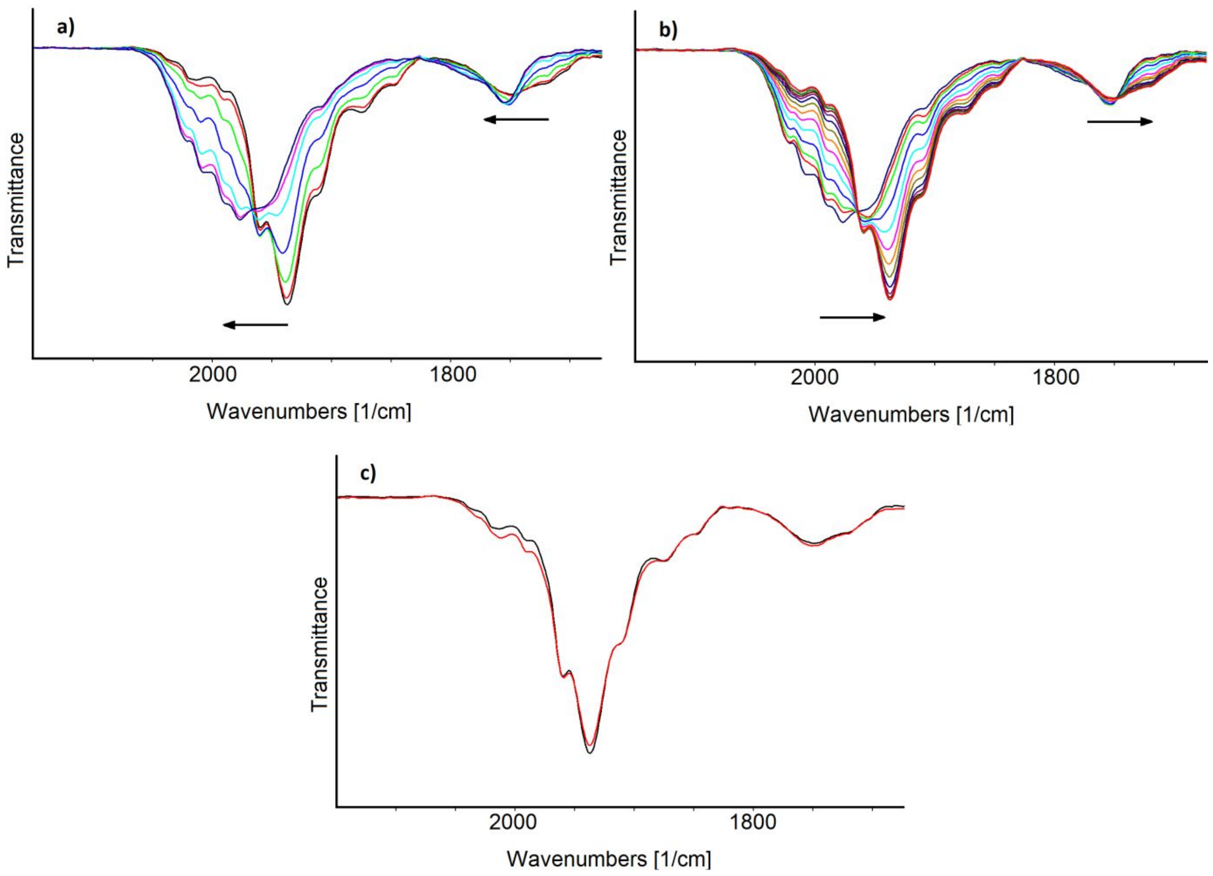

Figure S24. IR spectra of a CH_3CN solution of $[HRu_6C(CO)_{15}]^{3-}$ (**3**) recorded in an OTTLE cell during a) the progressive increase of the potential from –0.1 to +0.4 V; b) the reduction back-scan of the potential from +0.4 to –0.4 V, *vs* Ag pseudo reference electrode (scan rate 1 mV sec^{-1}); c) comparison between the initial spectrum (black line) of a) and the final spectrum (red line) of b). $[N^{n}Bu_4][PF_6]$ (0.1 mol dm^{-3}) as the supporting electrolyte. The absorptions of the solvent and supporting electrolyte have been subtracted.

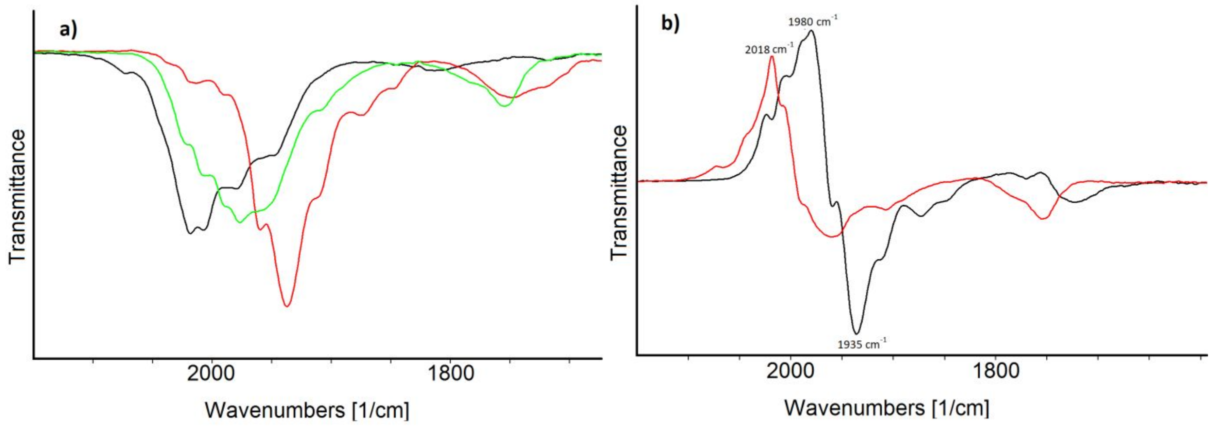

Figure S25. IR spectra of a CH_3CN solution of $[HRu_6C(CO)_{15}]^{3-}$ (**3**) recorded in an OTTLE cell a) at the potential of: –0.1 V (red line), +0.4 V (green line) and +0.7 V (black line); b) black line: difference spectrum between the red and the green spectra of part a) and red line: difference spectrum between the green and the black spectra of part a). $[N^{n}Bu_4][PF_6]$ (0.1 mol dm^{-3}) as the supporting electrolyte. The absorptions of the solvent and supporting electrolyte have been subtracted.

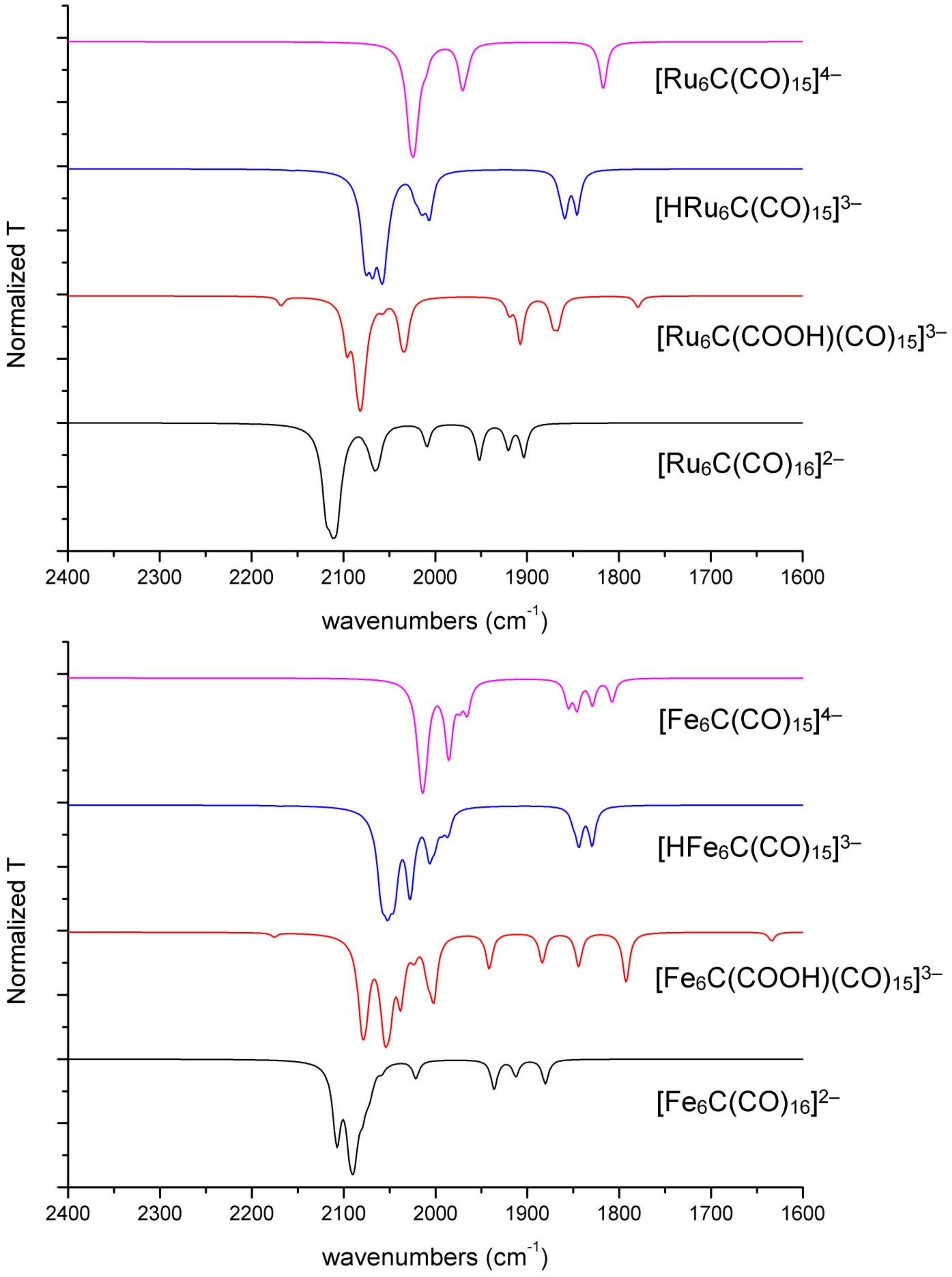

Figure S26. Unscaled simulated IR spectra of $[M_6C(CO)_{16}]^{2-}$, $[M_6C(COOH)(CO)_{15}]^{3-}$, $[HM_6C(CO)_{15}]^{3-}$ and $[M_6C(CO)_{15}]^{4-}$ (M = Fe, Ru). Lorentzian broadening functions, FWHM = 8 cm^{-1}.

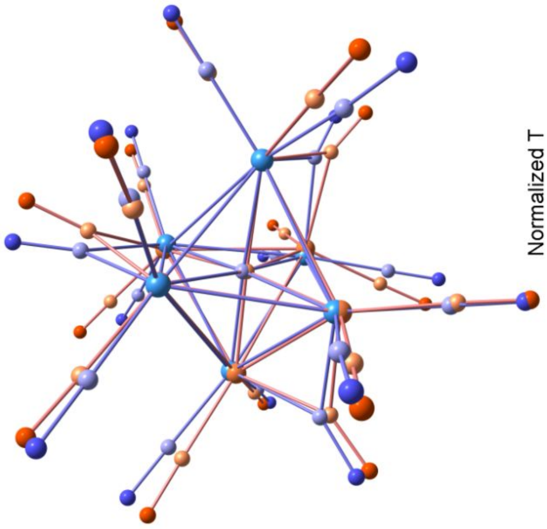

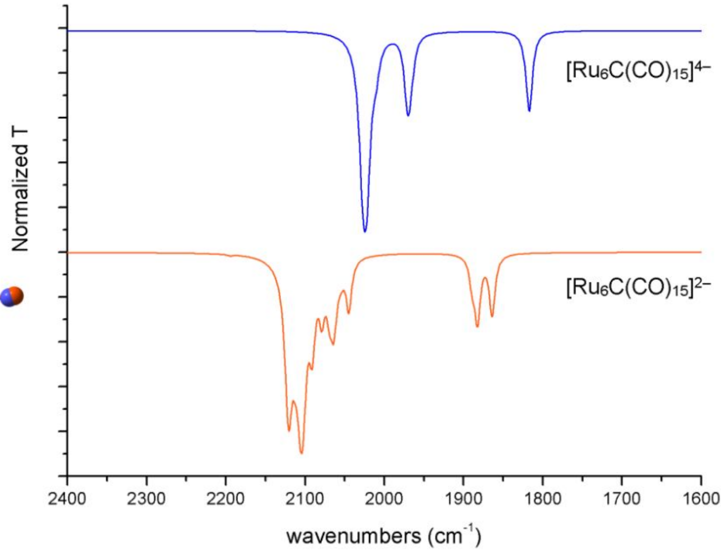

Figure S27. Superposition of the DFT-optimized structures of $[Ru_6C(CO)_{15}]^{2-}$ (**9**) (orange tones) and $[Ru_6C(CO)_{15}]^{4-}$ (**2**) (blue tones) and simulated IR spectra (Lorentzian broadening functions, FWHM = 8 cm^{-1}).

Table S1. AIM data for the Ru-carbide bonds calculated at (3,-1) BCPs (ρ = electron density; V = potential energy density; E = energy density; $\nabla^2\rho$ = Laplacian of electron density) and Hirshfeld charges of the atoms composing the $\{Ru_6C\}$ cores in $[Ru_6C(CO)_{15}]^{n-}$ (n = 2, 4).

**Cluster charge**	ρ (e Å^{-3})	**V** (hartree Å^{-3})	**E**[a] (hartree Å^{-3})	$\nabla^2\rho$[a] (e Å^{-5})	**Ru charge** (average, a.u.)	**C charge** (carbide, a.u.)
-2	0.802	-1.050	-0.345	5.142	0.097	-0.395
-4	0.816	-1.080	-0.356	5.274	0.049	-0.391

[a] The negative values of the E and the positive values of $\nabla^2\rho$ are in line with Bianchi's definition of dative bonds [Lepetit C.; Fau P.; Fajerweg K.; Kahn M. L.; Silvi B. Topological analysis of the metal-metal bond: A tutorial review. *Coord. Chem. Rev.* **2017**, *345*, 150-181].

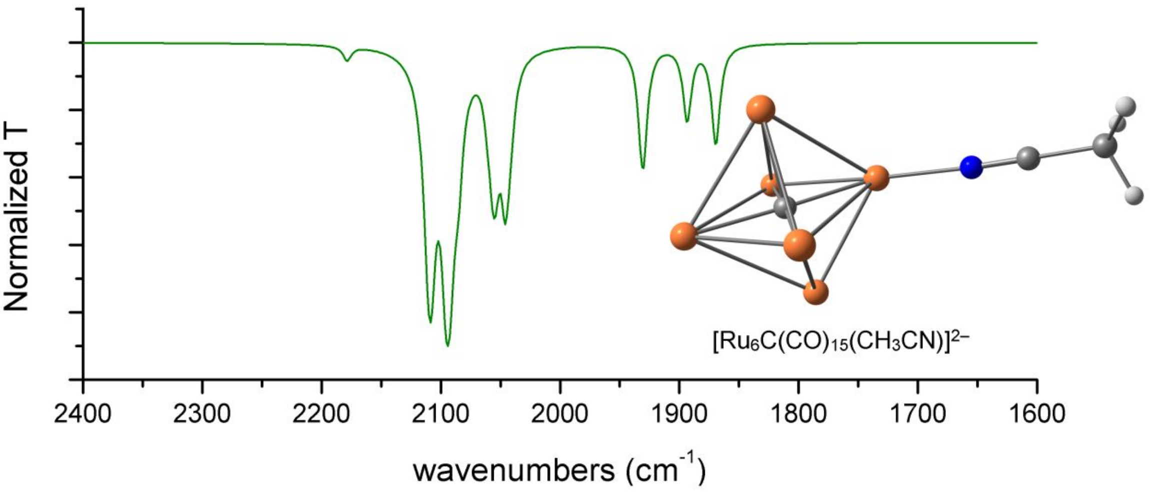

Figure S28. Simulated IR spectrum of $[Ru_6C(CO)_{15}(CH_3CN)]^{2-}$ (**5**) (Lorentzian broadening functions, FWHM = 8 cm^{-1}) and DFT-optimized structure. Color map: orange Ru; blue N; grey C; white H. Carbonyl ligands are omitted for clarity.

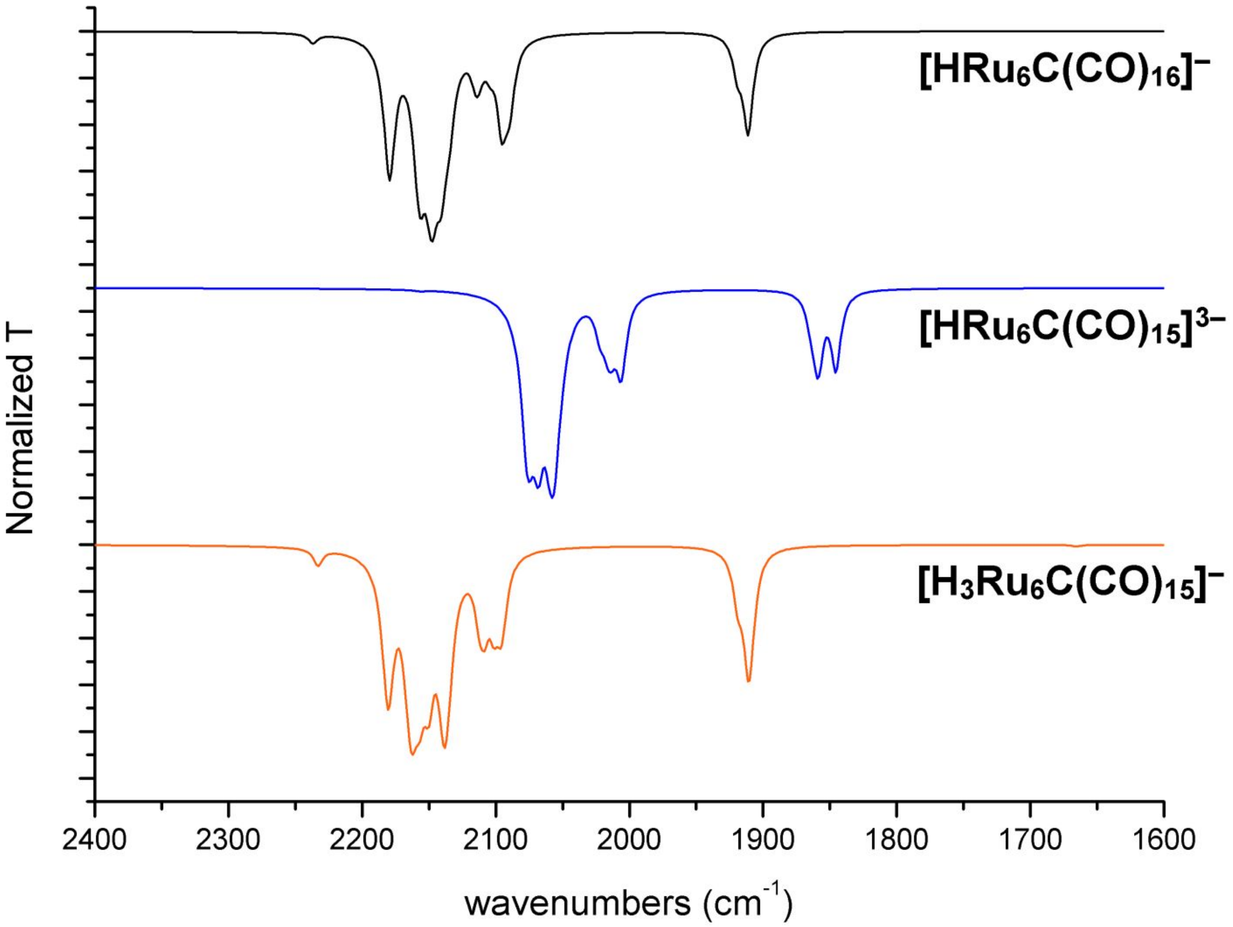

Figure S29. Simulated IR spectra of $[H_3Ru_6C(CO)_{15}]^{-}$ (**7**), $[HRu_6C(CO)_{15}]^{3-}$ (**3**) and $[HRu_6C(CO)_{16}]^{-}$ (**4**) (Lorentzian broadening functions, FWHM = 8 cm^{-1}).

Table S2. AIM data for the Ru-carbide bonds and Hirshfeld charges of atoms composing the $\{H_xRu_6C\}$ fragments in $[H_3Ru_6C(CO)_{15}]^-$ (**7**), $[HRu_6C(CO)_{15}]^{3-}$ (**3**) and $[HRu_6C(CO)_{16}]^-$ (**4**).

**Cluster**	ρ (e Å^{-3})	**V** (hartree Å^{-3})	**E** (hartree Å^{-3})	$\nabla^2\rho$ (e Å^{-5})	**Ru charge** (average, a.u.)	**C charge** (carbide, a.u.)	**H charge** (a.u.)
**7**	0.798	-1.035	-0.341	5.037	0.134	-0.385	-0.102
**3**	0.826	-1.095	-0.364	5.250	0.087	-0.390	-0.110
**4**	0.812	-1.073	-0.352	5.192	0.123	-0.386	-0.087

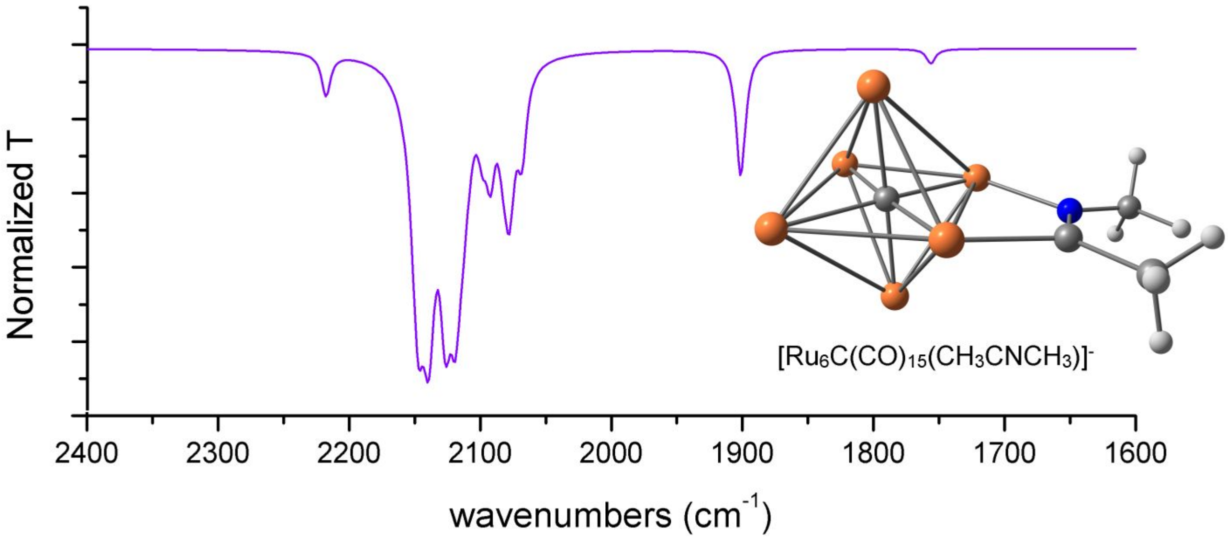

Figure S30. Simulated IR spectrum of $[Ru_6C(CO)_{15}(CH_3CNCH_3)]^-$ (**8**) (Lorentzian broadening functions, FWHM = 8 cm^{-1}) and DFT-optimized structure. Color map: orange Ru; blue N; grey C; white H. Carbonyl ligands are omitted for clarity.

Table S3. AIM data for the Ru-carbide bonds and Hirshfeld charges of the atoms composing the $\{Ru_6C\}$ cores in $[Ru_6C(CO)_{15}(CH_3CN)]^{2-}$ (**5**) and $[Ru_6C(CO)_{15}(CH_3CNCH_3)]^-$ (**8**).

**Cluster**	ρ (e Å^{-3})	**V** (hartree Å^{-3})	**E** (hartree Å^{-3})	$\nabla^2\rho$ (e Å^{-5})	**Ru charge** (average, a.u.)	**C charge** (carbide, a.u.)
**5**	0.801	-1.057	-0.343	5.208	0.084	-0.387
**8**	0.821	-1.090	-0.359	5.258	0.107	-0.387

Table S4. Crystal data and experimental details for $[NEt_4]_4[Ru_6C(CO)_{15}]\cdot CH_3CN$, $[NEt_4]_3[HRu_6C(CO)_{15}]$, $[NEt_4][H_3Ru_6C(CO)_{15}]$, $[NEt_4][HRu_6C(CO)_{16}]$, $[NEt_4][Ru_6C(CO)_{15}(CH_3CNCH_3)]\cdot solv$, $[NEt_4]_2[Ru_6C(CO)_{15}(CH_3CN)]$, $[NEt_4][RuCl_3(CO)_2(CH_3CN)_2]$.

	**$[NEt_4]_4[Ru_6C(CO)_{15}]\cdot CH_3CN$**	**$[NEt_4]_3[HRu_6C(CO)_{15}]$**	**$[NEt_4][H_3Ru_6C(CO)_{15}]$**
Formula	$C_{50}H_{83}N_5O_{15}Ru_6$	$C_{40}H_{61}N_3O_{15}Ru_6$	$C_{24}H_{23}NO_{15}Ru_6$
Fw	1600.63	1430.33	1171.85
T, K	100(2)	100(2)	100(2)
λ, Å	0.71073	0.71073	0.71073
Crystal system	Trigonal	Triclinic	Monoclinic
Space Group	$P3_221$	$P\bar{1}$	$P2_1/c$
a, Å	12.3936(5)	12.3553(14)	9.5100(7)
b, Å	12.3936(5)	12.8583(14)	12.7776(9)
c, Å	33.4743(14)	30.818(3)	26.523(2)
α, °	90	88.757(4)	90
β, °	90	89.251(4)	93.390(3)
γ, °	120	85.614(4)	90
Cell Volume, Å^3	4452.8(4)	4880.2(9)	3217.3(4)
Z	3	4	4
D_c, g cm^{-3}	1.791	1.947	2.419
μ, mm^{-1}	1.553	1.876	2.814
F(000)	2406	2824	2232
Crystal size, mm	0.24×0.22×0.19	0.16×0.11×0.10	0.16×0.12×0.09
θ limits, °	1.897-25.014	1.653-25.027	1.770–25.999
Index ranges	$-14 \le h \le 14$<br>$-14 \le k \le 14$<br>$-39 \le l \le 39$	$-14 \le h \le 14$<br>$-15 \le k \le 15$<br>$-36 \le l \le 36$	$-11 \le h \le 11$<br>$-15 \le k \le 15$<br>$-32 \le l \le 32$
Reflections collected	56103	78726	38575
Independent reflections	5255 [R_{int} = 0.0422]	17159 [R_{int} = 0.1707]	6159 [R_{int} = 0.0847]
Completeness to θ max	99.8%	99.5%	98.0%
Data / restraints / parameters	5255 / 258 / 357	17159 / 937 / 1160	6159 / 57 / 428
Goodness on fit on F^2	1.174	1.166	1.229
R_1 (I > 2σ(I))	0.0958	0.1454	0.0646

wR_2 (all data)	0.2087	0.3548	0.1275
Largest diff. peak and hole, e Å^{-3}	2.928 / -1.587	3.416 / -3.306	1.650 / –1.164

	**[NEt$_4$] [RuCl$_3$(CO)$_2$(CH$_3$CN)$_2$]**	**[NEt$_4$] [HRu$_6$C(CO)$_{16}$]**	**[NEt$_4$] [Ru$_6$C(CO)$_{15}$(CH$_3$CNCH$_3$)]· solv**	**[NEt$_4$]$_2$ [Ru$_6$C(CO)$_{15}$(CH$_3$CN)]**
Formula	$C_{12}H_{23}Cl_3N_2O_2Ru$	$C_{25}H_{21}NO_{16}Ru_6$	$C_{27}H_{26}N_2O_{15}Ru_6$	$C_{34}H_{43}N_3O_{15}Ru_6$
Fw	434.74	1197.85	1224.92	1340.13
T, K	100(2)	100(2)	100(2)	100(2)
λ, Å	0.71073	0.71073	0.71073	0.71073
Crystal system	Monoclinic	Monoclinic	Orthorhombic	Orthorhombic
Space Group	$P2_1/n$	$C2/c$	$Pbcn$	$P2_12_12_1$
a, Å	15.4077(12)	22.5802(16)	34.050(4)	11.5430(5)
b, Å	7.6274(6)	14.4388(10)	11.6549(12)	18.1372(7)
c, Å	15.8250(12))	12.1661(9)	19.642(2)	20.4877(9)
α, °	90	90	90	90
β, °	102.932(2)	122.068(2)	90	90
γ, °	90	90	90	90
Cell Volume, Å^3	1812.6(2)	3361.3(4)	7795.0(14)	4289.3(3)
Z	4	4	8	4
D_c, g cm^{-3}	1.593	2.367	2.088	2.075
μ, mm^{-1}	1.309	2.699	2.329	2.127
F(000)	880	2280	4688	2608
Crystal size, mm	0.15×0.14×0.11	0.16×0.15×0.11	0.19×0.13×0.09	0.13×0.10×0.08
θ limits, °	1.668–28.000	2.129–26.000	1.847–25.096	1.988–25.998
Index ranges	$-20 \le h \le 20$<br>$-10 \le k \le 10$<br>$-20 \le l \le 20$	$-27 \le h \le 27$<br>$-17 \le k \le 17$<br>$-14 \le l \le 14$	$-40 \le h \le 40$<br>$-13 \le k \le 13$<br>$-23 \le l \le 23$	$-14 \le h \le 14$<br>$-22 \le k \le 22$<br>$-25 \le l \le 25$
Reflections collected	31783	22635	72164	59852
Independent reflections	4357 [R_{int} = 0.0656]	3305 [R_{int} = 0.0366]	6891 [R_{int} = 0.0952]	8424 [R_{int} = 0.0476]
Completeness to θ max	99.9%	99.8%	99.4%	100.0%

Data / restraints / parameters	4357 / 0 / 186	3305 / 154 / 286	6891 / 222 / 457	8424 / 0 / 533
Goodness on fit on F^2	1.118	1.153	1.283	1.162
R_1 ($I > 2\sigma(I)$)	0.0274	0.0297	0.1098	0.0272
wR_2 (all data)	0.0629	0.0678	0.2469	0.0536
Largest diff. peak and hole, e Å^{-3}	0.936 / –0.401	1.343 / –0.800	3.032 / –3.351	0.727 / –0.687
